# Bidirectional relationships between intolerance of uncertainty and generalized anxiety among adolescents: insights from cross-lagged panel network analysis

**DOI:** 10.1186/s13034-025-00912-6

**Published:** 2025-05-14

**Authors:** Haoxian Ye, Yunyi Li, Yike Huang, Yiming Zhang, Jiaxiong Zhang, Jiaqi Wang, Keying Liu, Yuyi Yao, Xinyu Shi, Yijia Liu, Fang Fan

**Affiliations:** 1 School of Psychology, Centre for Studies of Psychological Applications, Guangdong Key Laboratory of Mental Health and Cognitive Science, Guangdong Emergency Response Technology Research Center for Psychological Assistance in Emergencies, South China Normal University, Guangzhou, China; 2 Key Laboratory of Brain, Cognition and Education Sciences, Philosophy and Social Science Laboratory of Reading and Development in Children and Adolescents (South China Normal University), Ministry of Education, Guangzhou, China; 3 School of Psychology, South China Normal University, Shipai Road, Guangzhou, 510631 China

**Keywords:** Adolescents, Bidirectional, Intolerance of uncertainty, Generalized anxiety, Cross-lagged panel network

## Abstract

**Background:**

Intolerance of uncertainty (IU) has received increasing attention for its role in the development and maintenance of generalized anxiety. However, little is known about the temporal and causal relationships between IU and generalized anxiety, particularly in adolescents. Furthermore, much of the existing literature treats IU and generalized anxiety as unidimensional constructs, limiting a detailed understanding of their internal elements and specific symptom interactions. To address the gaps, this study employed a cross-lagged panel network (CLPN) approach to examine the temporal interactions and predictive relationships between IU elements and generalized anxiety symptoms.

**Methods:**

A sample of 7,434 nonclinical adolescents (mean _age_ = 15.33 years, range = 11–19 years, 50.6% girls) completed the Intolerance of Uncertainty Scale (Short Form) for Children (IUSC-12) and the Generalized Anxiety Disorder Scale (GAD-7) across two waves, six months apart. Data was analyzed using the CLPN approach.

**Results:**

Bidirectional predictive relationships were found between IU elements and generalized anxiety symptoms, with generalized anxiety symptoms more frequently predicting IU elements. The generalized anxiety symptom named “nervousness” was the strongest predictor of increases in both IU elements and other generalized anxiety symptoms over time, while the IU elements named “frustration” and “work with hindrance” were the strongest predictors of future generalized anxiety symptoms.

**Conclusions:**

This study provides new insights into the reciprocal relationships between IU and generalized anxiety among adolescents, highlighting the complex interplay between vulnerability and mental health problems. By identifying key IU elements and generalized anxiety symptoms that drive these relationships, the findings contribute to a more nuanced understanding of adolescent psychopathology and inform targeted interventions.

**Supplementary Information:**

The online version contains supplementary material available at 10.1186/s13034-025-00912-6.

## Introduction

Generalized anxiety is one of the most prevalent mental health conditions among adolescents worldwide [1], with onset typically occurring in childhood and adolescence [2]. Characterized by persistent, excessive, and uncontrollable worry about various aspects of life [3], generalized anxiety frequently co-occurs with other mental health problems [4] and has a profound impact on adolescents, affecting their academic performance, social relationships, and overall quality of life [5]. While cognitive behavioral therapy (CBT) is an effective treatment for adolescent generalized anxiety, recent data indicate that nearly 40% of adolescents retain an anxiety diagnosis even after completing CBT [6]. This underscores the need for further investigation into the factors underlying the development and maintenance of adolescent generalized anxiety to refine and enhance therapeutic strategies.

Intolerance of uncertainty (IU), a dispositional inability to tolerate the distress caused by uncertainty, has recently been identified as a key vulnerability associated with generalized anxiety in adolescents [7]. However, the temporal and causal nature of their relationship remains unclear, particularly at a fine-grained level, examining specific elements of IU and symptoms of generalized anxiety. To address these gaps, this study applied the cross-lagged panel network (CLPN) approach to investigate the directionality of the relationships between IU and generalized anxiety. By identifying the core elements and symptoms driving their longitudinal interactions, we aimed to deepen our understanding of the dynamic interplay between IU and generalized anxiety in adolescents.

### Role of IU in generalized anxiety

Since generalized anxiety is inherently tied to uncertainty due to its focus on potential future threats [8, 9], adolescents who perceive and interpret uncertainty as threatening are more likely to experience distress and face an elevated risk of developing generalized anxiety [10]. This vulnerability is encapsulated by the construct of IU, defined as a dispositional inability to endure the distress triggered and sustained by perceived uncertainty [11]. According to the Intolerance of Uncertainty Model (IUM), IU acts as a higher-order vulnerability factor for excessive and persistent worry by predisposing individuals to maintain positive beliefs about worry, engage in cognitive avoidance, and adopt a negative problem orientation [12], which collectively heighten susceptibility to the full spectrum of generalized anxiety symptoms [13]. Empirical evidence aligns with this model. Meta-analyses have revealed strong associations between IU and generalized anxiety [14], while experimental studies demonstrate that manipulating IU results in parallel changes in worry (the core feature of generalized anxiety) [15, 16]. Furthermore, treatment research supports the efficacy of IU-focused interventions in reducing generalized anxiety symptoms [17, 18]. These findings consistently highlight the central role of IU in explaining generalized anxiety, emphasizing the importance of addressing IU within the context of CBT for anxiety disorders.

### Relationships between IU and generalized anxiety among adolescents

While increasing evidence highlights IU as a key vulnerability for generalized anxiety [14, 19, 20], much of this research has been conducted with adults, raising concerns about the direct applicability of these findings to adolescents. Although previous studies have reported strong associations between IU and generalized anxiety in adolescents [21, 22], these findings are predominantly based on cross-sectional data, as noted in a recent meta-analysis [7], which limits our understanding of whether IU plays a causal role in the development and maintenance of generalized anxiety in adolescents. Addressing this gap is critical for evaluating the clinical utility of IU-targeted interventions, as their effectiveness depends on IU serving as a causal rather than an epiphenomenal factor in adolescent generalized anxiety [23].

To date, only two studies have longitudinally examined the relationship between IU and generalized anxiety in adolescents. In the first study, using a ten-wave, five-year cohort design, researchers found that IU and worry (the hallmark of generalized anxiety) shared a bidirectional and reciprocal relationship, exerting equal mutual influence over time [24]. This result challenges the traditional unidirectional perspective proposed by the Intolerance of Uncertainty Model (IUM) [10] and suggests that the relationship between IU and psychopathology may be more dynamic than previously assumed. However, the study’s focus on worry rather than the full symptomatology of generalized anxiety potentially limit the generalizability of its findings. In another recent study, Marchetti et al. (2025) employed necessary condition analysis and found that IU was a necessary condition for the development of generalized anxiety symptoms in adolescents. This finding extends the traditional view of IU as merely a predictor [7], and instead suggests it as a prerequisite for adolescent generalized anxiety symptoms. Taken together, this emerging body of longitudinal research preliminarily demonstrates the central role of IU in adolescent generalized anxiety. However, further investigation is needed to clarify the temporal directionality and potential reciprocal influences between IU and generalized anxiety symptoms—questions that remain unresolved in the current literature.

### Potential bidirectional relationships between IU and generalized anxiety among adolescents

Several theoretical and empirical reasons suggest that IU and generalized anxiety may have bidirectional relationships among adolescents. For instance, as adolescence is a critical developmental period marked by significant neural plasticity and the formation of cognitive patterns [25], key cognitive processes necessary for reasoning about uncertainty, such as introspective awareness [26, 27] and meta-cognitive skills [28, 29], continue to develop during this time. Frequent experiences of generalized anxiety symptoms may disrupt this cognitive maturation by causing physiological, cognitive, and behavioral changes, which may impair the normal development of adolescents’ ability to tolerate uncertainty [7]. In turn, a diminished capacity to endure uncertainty heightens vulnerability to generalized anxiety symptoms [10], potentially creating a cycle where IU drives the development of generalized anxiety and generalized anxiety exacerbates IU. In line with this theoretical framework, empirical studies have documented longitudinal bidirectional relationships between IU and psychiatric symptom severity in various populations, including refugees [30] and treatment-seeking veterans [31], while a treatment study has observed concurrent bidirectional predictive relationships between IU and worry severity in clinical adults undergoing CBT for generalized anxiety [32]. However, these studies have focused on vulnerable adults in disadvantaged or clinical settings, leaving it unclear whether such bidirectional relationships exist in nonclinical adolescents. Longitudinal research is thus needed to determine whether IU functions both as a precursor to and a consequence of generalized anxiety in nonclinical adolescents. Such insights could help refine and optimize IU-focused psychological interventions for adolescents with generalized anxiety [23].

### Strengths for adopting the network approach

To date, most cross-sectional and longitudinal studies on IU and generalized anxiety in adolescents have traditionally conceptualized both constructs as latent variables, operationalized as composite scores of their respective scales. While useful, this approach oversimplifies the complexity of their relationship, given that generalized anxiety is a heterogeneous syndrome characterized by diverse affective, cognitive, and somatic symptoms [33], and IU encompasses a range of cognitive, emotional, and behavioral responses to uncertainty [34]. To better understand the nuanced pathways linking IU elements and generalized anxiety symptoms, it is essential to move beyond broad categories and examine these constructs at the symptom and element levels.

The network approach provides a promising framework for studying the intricate interconnections between individual symptoms of mental disorders and elements of their risk factors [35]. Unlike traditional models that view mental disorders as resulting from a single latent cause, the network approach conceptualizes disorders as emerging from complex interactions among their constituent symptoms [35]. Additionally, this framework allows researchers to incorporate risk factors (as well as their internal elements) into the symptom networks, offering a more intuitive understanding of how risk factors and disorders interact [36]. In these networks, symptoms and risk factors (as well as their internal elements) are represented as nodes, while their pairwise interactions are depicted as edges. This enables identification of the risk factors that contribute most significantly to symptoms and the specific pathways mediating these effects [37, 38]. Furthermore, metrics such as centrality and predictability can quantify the importance and controllability of nodes [35, 39], providing actionable targets for interventions to reduce overall network connectivity between risk factors and disorders.

Empirical evidence supports the feasibility of this approach. For example, Ren et al. (2021) examined the network of IU and generalized anxiety in 624 nonclinical undergraduate students (aged 18–25), which demonstrated that adding IU elements as nodes in generalized anxiety symptoms networks is both practically and theoretically valuable. Their findings highlighted a strong connection between the IU element “frustration” and the generalized anxiety symptom “excessive worry,” with both nodes playing critical roles in activating and maintaining the network [40]. However, as their study used cross-sectional and undirected networks, it remains unknown the prospectively predictive effects of these so-called central nodes, as well as the temporal and causal relationships between IU and generalized anxiety over time. The recent cross-lagged panel network (CLPN) approach offers a promising solution for addressing these limitations. By simultaneously estimating autoregressive effects (how a variable predicts itself over time) and cross-lagged effects (how variables predict one another over time), the CLPN approach allows for the identification of longitudinal predictive pathways between IU elements and generalized anxiety symptoms [41]. Beyond detecting potentially causal paths, this method also calculates more specific centrality indices than cross-sectional networks, identifying which nodes are most central in predicting others and being predicted themselves. This provides valuable insights for advancing theoretical understanding and informing targeted intervention strategies for adolescent mental health.

### Current study

Adolescence is a critical developmental stage characterized by heightened vulnerability to mental health problems due to significant biological, social, and psychological changes [42]. Identifying the relationships between common mental health issues and their risk factors during this period is thus essential for developing effective prevention and intervention strategies. To this end, this study aimed to use the CLPN approach to examine the longitudinal interactions between IU elements and generalized anxiety symptoms in adolescents. Specifically, our objectives were (1) to estimate the cross-lagged relationships between IU elements and generalized anxiety symptoms, providing preliminary directional insights into their relationships, and (2) to calculate centrality indices to identify the IU elements or generalized anxiety symptoms that play the most predictive or influential roles in driving the development of IU and generalized anxiety over time. Given the complexity of network analysis, we did not propose specific hypotheses. We expected our explorations would reveal potential causal pathways in the dynamic relationship between IU and generalized anxiety, enhancing our understanding of the role of IU in adolescent generalized anxiety and informing more effective prevention, intervention, and treatment strategies for adolescents.

## Methods

### Participants

Data were drawn from an ongoing longitudinal adolescent mental health project, using cluster sampling methods to recruit participants from five randomly selected middle and high schools in a western city in Guangdong, China. The study adhered to the principles of the Helsinki Declaration (2013 revision) and received approval from the Ethics Committee of South China Normal University (SCNU-PSY-2024-119). For the current analysis, we utilized data from the first (March 4–24, 2024) and second (September 3–30, 2024) survey waves, hereafter referred to as Time 1 (T1) and Time 2 (T2). Exclusion criteria at each wave were (1) incorrect identity information (e.g., mismatched student IDs), (2) unreasonably short response times (e.g., less than one second per item), (3) failure to answer attention check items correctly (e.g., instructions to select a specific response), (4) patterned or inconsistent responses, and (5) a history of mental health illness (ensuring the nonclinical nature of the sample). Following these criteria, 9,127 students provided valid responses at T1, with 7,434 of them further providing valid responses at T2 (retention rate = 81.5%). The reasons for attrition included transferring to other schools and being absent from school at the time of the assessment or for other reasons. Independent t-tests were conducted to assess attrition effects by comparing key variables between students who completed both waves and those who participated only at T1. No significant differences were observed in generalized anxiety symptom severity scores (3.35 vs. 3.32, *p* = 0.774) or IU level scores (30.26 vs. 29.73, *p* = 0.080).

### Procedures

Data collection was conducted online during regular school hours, with support from the local education bureau and school mental health departments. Surveys were administered in school computer rooms under standardized conditions. Before starting, researchers emphasized that participation was voluntary, responses were confidential, and the data would be used exclusively for scientific purposes. Participants completed the web-based survey via a secure online platform using an anonymous student ID. As the platform required all items to be completed prior to submission, there were no missing data for any of the variables included in the present analyses. Electronic informed consent was obtained from all participants and their caregivers prior to data collection. To ensure support for participants, psychological crisis intervention training was provided to school psychology teachers to help them identify high-risk students and provide timely assistance. Additionally, a free psychological distress hotline was made available to all participants and their caregivers, encouraging them to seek guidance or support as needed.

### Measures

#### Intolerance of uncertainty (IU)

The Chinese version of the Intolerance of Uncertainty Scale (Short Form) for Children (IUSC-12) was used to evaluate adolescents’ levels of IU via their emotional, cognitive, and behavioral maladaptive responses to uncertainty [43]. Participants rated each item on a 5-point Likert scale ranging from 1 (not at all) to 5 (very much), with total scores ranging from 12 to 60. Higher scores indicate greater levels of IU. The Chinese version of the IUSC-12 has demonstrated strong reliability and validity in Chinese adolescents [44] and has been used in adolescent network research [34]. Cronbach’s alpha was 0.917 at T1 and 0.961 at T2.

#### Generalized anxiety symptoms

The 7-item Generalized Anxiety Disorder Scale (GAD-7), a validated self-report tool based on DSM criteria [45], was used to assess the severity of generalized anxiety symptoms over the past two weeks [33]. Each item was rated on a 4-point Likert scale from 0 (not at all) to 3 (nearly every day), with higher scores indicating greater anxiety severity. The Chinese version of the GAD-7 has shown strong psychometric properties in Chinese adolescents [46, 47] and has been widely used in adolescent anxiety-related network research [48, 49]. A cutoff score of 10 has been recommended for identifying probable clinical anxiety [50]. Cronbach’s alpha was 0.945 at T1 and 0.961 at T2.

#### Covariates

1. Sociodemographic characteristics. A self-report questionnaire collected data on age, sex, ethnicity, family income, parental marital status, immigrant status, single-child status, family history of psychiatric illness, left-behind status, and chronic physical illness at T1. These variables were included as covariates due to their potential influence on IU and generalized anxiety [22].
2. Negative life events (NLEs). The Adolescents Self-Rating Life Events Checklist (ASLEC) [51] was used to assess the severity of NLEs experienced between T1 and T2. The ASLEC includes 27 items covering six domains: physical health problems, personal loss, interpersonal conflicts, academic pressure, being punished, and other stressors. Items were rated on a 5-point Likert scale from 1 (not at all) to 5 (extremely severe). Cronbach’s alpha was 0.967 at T2.

### Data analysis

Each item of the IUSC-12 was treated as an IU element, and each item of the GAD-7 was treated as a generalized anxiety symptom. Descriptive statistics and paired t-tests for IU elements and generalized anxiety symptoms at T1 and T2 were conducted using SPSS 23.0. Although none of them violated normality based on skewness > 2 and/or kurtosis > 7 [52], a nonparanormal transformation [53] was applied to relax the normality assumption, following established guidelines for psychological network analyses [54].

All network analyses were conducted in R 4.3.0. In line with previous CLPN studies [55–57], we utilized the netSimulator function in the “bootnet” package [58] to estimate network power. Three properties were employed to determine the appropriate sample size based on the anticipated network model and the established network structure [54]: (1) sensitivity, also known as the true-positive rate, refers to the proportion of edges present in the true network that are detected as significant in the estimated network; (2) specificity, or the true-negative rate, is the proportion of missing edges in the true network that are correctly identified as absent in the estimated network; and (3) correlation between edge weights in the true network and the estimated network. A sample size is considered adequate if all three properties exhibit sufficiently high values, as indicated in previous research on network power analysis [54]. After the power analysis, the CLPN was estimated using LASSO regularization with 10-fold cross-validation via the “glmnet” package [59]. Sociodemographic characteristics at T1 and NLEs from T1 to T2 were included as covariates, given their potential influence on adolescent IU [22, 60]. The estimated CLPN was then visualized using the “qgraph” package [61]. To identify the most influential elements and symptoms in the network, we calculated two centrality indices (in-prediction and out-prediction) through the R-package “lavaan” [62]. The in-prediction is the extent to which each node is influenced by other nodes in the network, and out-prediction is the extent to which each node predicts other variables in the network. The higher the in-prediction, the more it is influenced by other nodes, while the higher the out-prediction, the more it influences the other nodes. In line with previous CLPN studies on risk factors and mental disorders [56, 63, 64], we calculated the in-prediction and out-prediction for cross-lagged (excluding autoregressive path of the node of interest) and cross-construct (excluding autoregressive path and paths within the same construct). The cross-lagged analysis examines the proportion of variance accounted for by all other variables at the previous measurement time, while the cross-construct in-prediction examines the proportion of variance accounted for by all variables that belong to a different construct at the previous measurement time. Finally, the accuracy and stability of the CLPN were assessed using 5,000 nonparametric and 5,000 case-drop bootstraps via the “bootnet” package [58]. Due to page limitations, further methodological details of the network analyses are available in *Supplemental Materials*.

## Results

### Sample characteristics

The study included 7,434 nonclinical adolescents with an average baseline age of 15.33 years (SD = 1.57 years, ranged from 11 to 19 years), of whom 50.6% were girls. Sociodemographic information, and IU and generalized anxiety details are presented in Tables 1 and 2, respectively. Descriptive characteristics and paired t-tests results of each IU element and generalized anxiety symptom were illustrated in Table 3. Table 1 shows mean IU scores of 30.26 (SD = 11.16) at T1 and 31.18 (SD = 10.61) at T2. These scores are comparable to the mean IU score (M = 34.47, SD = 9.73) reported in a validation study of the Chinese version of the IUSC-12 conducted with nonclinical Chinese adolescents [44]. Similarly, the mean score of generalized anxiety symptoms was 3.35 (SD = 4.22) at T1 and 3.15 (SD = 4.04) at T2, which aligns with the mean scores of generalized anxiety symptoms ranging from 2.25 to 4.76 reported in a large sample of nonclinical Chinese adolescents aged 10–17 years during the validation of the Chinese version of the GAD-7 [47]. These comparisons suggest that the current sample falls within the normative range for nonclinical adolescent populations on both IU and generalized anxiety measures.

**Table 1:** Descriptive statistics of the current sample (*n* = 7434)

Characteristics		*n*	%
Age^a^ [year, mean (SD)]	15.33 (1.57)		
Sex	Boys	3670	49.4
	Girls	3764	50.6
Ethnicity	Han^b^	7397	99.5
	Others	37	0.5
Parental martial status	Married	6767	91.0
	Not current married^c^	667	9.0
Immigrants’ second generation	Yes	619	8.3
Single-child status	Yes	354	4.8
Left-behind status^d^	Yes	1463	19.7
Chronic physical illness^e^	Yes	133	1.8
Family history of psychiatric illness	Yes	81	1.1
Family monthly incomes	< 6000	2456	33.0
	6000–12,000	1907	25.7
	12,000–18,000	372	5.0
	18,000–24,000	111	1.5
	> 24,000	144	1.9
	Unknown	2444	32.9
Negative life events [mean (SD)]	29.66 (13.97)		
IU levels at T1 [mean (SD)]	30.26 (11.16)		
IU levels at T2 [mean (SD)]	31.18 (10.61)		
Generalized anxiety symptoms severity at T1 [mean (SD)]	3.35 (4.22)		
Generalized anxiety symptoms severity at T2 [mean (SD)]	3.15 (4.04)		

*IU* intolerance of uncertainty

^a^The range of age in the current sample was 11–19 years

^b^Han is the major ethnic group in China

^c^Not current married included separated, divorced, and widowed

^d^Live separately from one or both parents for more than 6 months

^e^Chronic physical conditions referred to having at least one of arthritis angina, asthma, diabetes, visual impairment, or hearing problems

**Table 2:** Item labels and contents

Scales	Items	Labels	Contents
GAD-7	GA1	Nervousness	Feeling nervous, anxious or on edge
	GA2	Uncontrollable worry	Not being able to stop or control worrying
	GA3	Excessive worry	Worrying too much about different things
	GA4	Trouble relaxing	Trouble relaxing
	GA5	Restlessness	Being so restless that it is hard to sit still
	GA6	Irritability	Becoming easily annoyed or irritable
	GA7	Feeling afraid	Feeling afraid as if something awful might happen
IUSC-12	IU1	Upset	Surprise events upset me greatly
	IU2	Frustration	It frustrates me not having all the information I need
	IU3	“Should think ahead” belief	One should always think ahead to avoid surprises
	IU4	Catastrophizing belief	Plans can be ruined by things you didn’t think would happen
	IU5	Obsessive thoughts	I always want to know what will happen to me in the future
	IU6	Aversive attitude	I don’t like being taken by surprise
	IU7	“Should prepare everything” belief	I should be able to prepare for everything in advance
	IU8	Live with discomfort	Not knowing what could happen keeps me from enjoying life
	IU9	Be paralysed	When it is time to do things, not knowing what could happen keeps me from acting
	IU10	Work with hindrance	When I am not sure of something I can’t work very well
	IU11	Stop actions	The smallest doubt can stop me from doing things
	IU12	Escape uncertainty	I must get away from all situations where I don’t know what will happen

*GAD-7* the 7-item Generalized Anxiety Disorder Scale,* IUSC-12* the Chinese version of the Intolerance of Uncertainty Scale (Short Form) for Children,* GA* generalized anxiety,* IU* intolerance of uncertainty

**Table 3:** Results of descriptive analyses and paired t-tests (*n* = 7434)

Nodes	T1				T2				t	*p*	Difference [95% CI]	Cohen’s d [95% CI]
	M	SD	Skewness	Kurtosis	M	SD	Skewness	Kurtosis				
GA1	1.57	0.73	1.27	1.47	1.55	0.72	1.28	1.49	− 2.19	0.028	− 0.02 [− 0.04, − 0.00]	− 0.03 [− 0.05, − 0.00]
GA2	1.46	0.70	1.56	2.24	1.44	0.66	1.53	2.27	− 3.36	< 0.001	− 0.03 [− 0.04, − 0.01]	− 0.04 [− 0.06, − 0.02]
GA3	1.54	0.74	1.38	1.63	1.50	0.70	1.40	1.80	− 4.21	< 0.001	− 0.04 [− 0.05, − 0.02]	− 0.05 [− 0.07, − 0.03]
GA4	1.48	0.70	1.48	1.99	1.45	0.67	1.51	2.18	− 2.98	0.003	− 0.03 [− 0.04, − 0.01]	− 0.03 [− 0.06, − 0.01]
GA5	1.36	0.63	1.86	3.53	1.35	0.60	1.84	3.55	− 2.32	0.020	− 0.02 [− 0.03, − 0.00]	− 0.03 [− 0.05, − 0.00]
GA6	1.54	0.74	1.38	1.65	1.50	0.70	1.40	1.82	− 4.12	< 0.001	− 0.04 [− 0.05, − 0.02]	− 0.05 [− 0.07, − 0.03]
GA7	1.40	0.67	1.87	3.50	1.36	0.61	1.86	3.73	− 4.90	< 0.001	− 0.04 [− 0.06, − 0.02]	− 0.06 [− 0.08, − 0.03]
IU1	2.61	1.18	0.04	− 1.11	2.69	1.15	− 0.09	− 1.04	5.10	< 0.001	0.08 [0.05, 0.11]	0.06 [0.04, 0.08]
IU2	2.36	1.10	0.35	− 0.76	2.47	1.06	0.12	− 0.83	7.26	< 0.001	0.10 [0.07, 0.13]	0.08 [0.06, 0.11]
IU3	2.70	1.21	− 0.01	− 1.11	2.81	1.12	− 0.20	− 0.87	6.55	< 0.001	0.11 [0.07, 0.14]	0.08 [0.05, 0.10]
IU4	2.65	1.22	0.06	− 1.13	2.66	1.13	− 0.03	− 0.99	0.44	0.660	0.01 [− 0.02, 0.04]	0.01 [− 0.02, 0.03]
IU5	2.79	1.27	− 0.06	− 1.20	2.78	1.19	− 0.10	− 1.05	− 0.92	0.355	− 0.01 [− 0.05, 0.02]	− 0.01 [− 0.03, 0.01]
IU6	2.59	1.18	0.11	− 1.05	2.72	1.14	− 0.10	− 0.99	8.42	< 0.001	0.13 [0.10, 0.16]	0.10 [0.07, 0.12]
IU7	2.85	1.21	− 0.18	− 1.05	2.92	1.11	− 0.33	− 0.75	4.42	< 0.001	0.07 [0.04, 0.11]	0.05 [0.03, 0.07]
IU8	2.24	1.07	0.47	− 0.58	2.36	1.03	0.25	− 0.67	8.55	< 0.001	0.12 [0.09, 0.15]	0.10 [0.08, 0.12]
IU9	2.26	1.04	0.39	− 0.66	2.35	1.00	0.22	− 0.67	6.43	< 0.001	0.09 [0.06, 0.11]	0.07 [0.05, 0.10]
IU10	2.58	1.19	0.11	− 1.11	2.63	1.13	0.01	− 0.98	3.51	< 0.001	0.05 [0.02, 0.08]	0.04 [0.02, 0.06]
IU11	2.41	1.14	0.31	− 0.89	2.48	1.07	0.14	− 0.87	4.71	< 0.001	0.07 [0.04, 0.10]	0.05 [0.03, 0.08]
IU12	2.21	1.04	0.48	− 0.49	2.33	1.00	0.25	− 0.61	8.23	< 0.001	0.11 [0.09, 0.14]	0.10 [0.07, 0.12]

*GA* generalized anxiety,* IU* intolerance of uncertainty

### Cross-lagged panel network

**Fig. 1:**
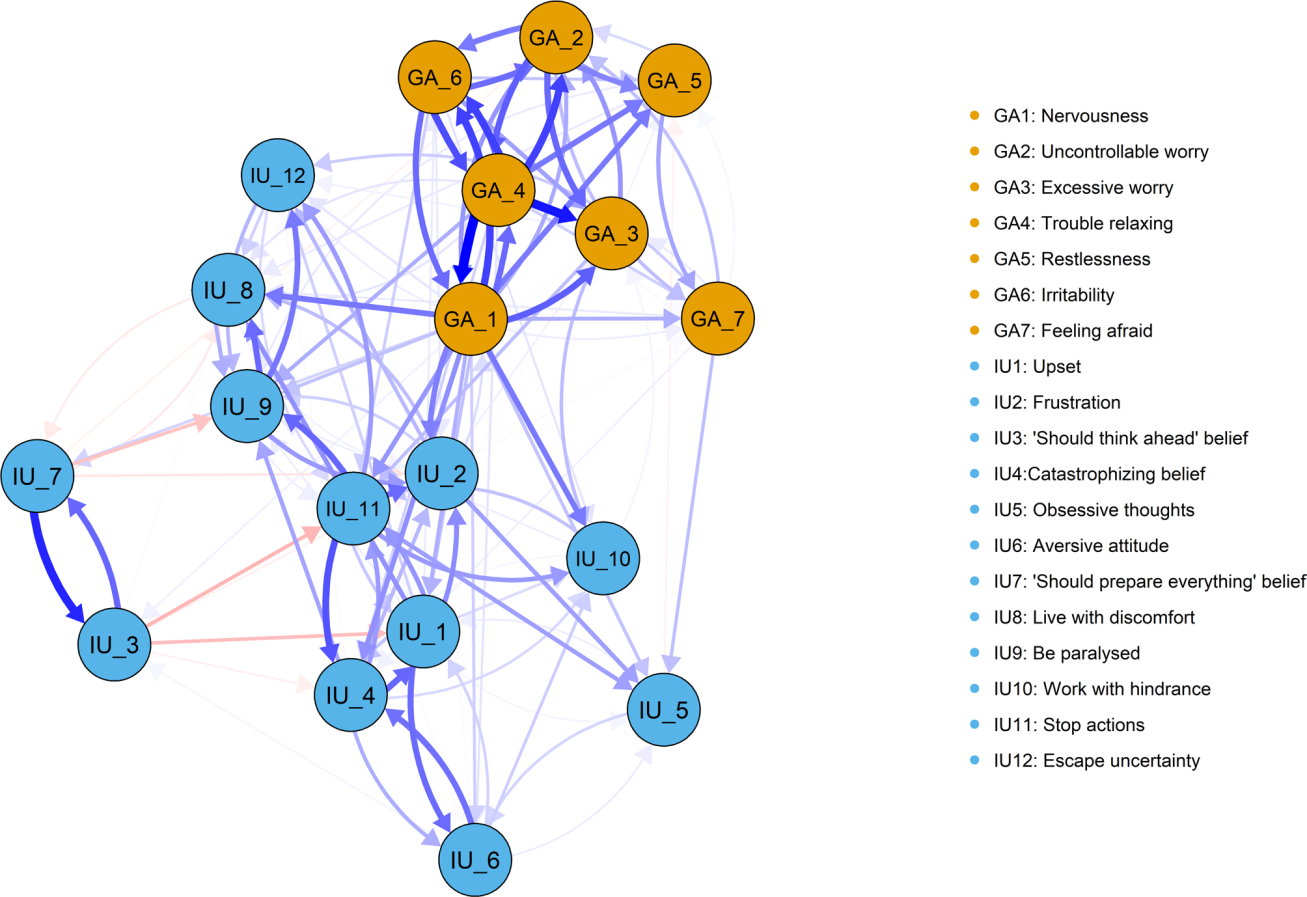
Plot for the CLPN (without auto-regressive edges). Edges depict cross-lagged effects and arrows indicate the direction of prediction. Edge thickness reflects the strength of the effects. Only the edges ≥ 0.014 would be illustrated in the plot

Based on the results of the power analysis (see Figure S1), a sample size of *n* = 7000 was deemed sufficient, with the correlation, sensitivity, and specificity of the network all exceeding 0.6, which aligns with the recommended standards for adequate power in network analysis [54]. All edge weights presented in the LASSO cross-lagged regression matrix are in Table S1. The autoregressive coefficients of each edge are shown in Figure S2. Given that the autoregressive edges (mean = 0.14) exhibited greater strength compared to the cross-lagged edges (mean = 0.014), we plotted the network structures without the autoregressive edges to highlight the cross-lagged effects most relevant to our study aims in Fig. 1, while the plot of network structures including the autoregressive edges is available in Figure S3. In line with the previous CLPN study [56], we set the threshold to be higher than the mean of all cross-lagged edge weights (0.014) to remove low-weight edges and retain the meaningful edges.

There were 223 non-zero cross-lagged edges in the CLPN, with 194 positive cross-lagged edges (87%). Specifically, a total of 33 positive connections in which IU elements predict generalized anxiety symptoms were found, while the top three strongest predict lines were: “frustration (IU2)→nervousness (GA1)”, “live with discomfort (IU8)→trouble relaxing (GA4)”, and “upset (IU1)→irritability (GA6)” (see Table S2). Conversely, a total of 52 positive connections in which generalized anxiety symptoms predict IU elements were found, while the top three predict lines comprised: “nervousness (GA1)→live with discomfort (IU8)”, “nervousness (GA1)→work with hindrance (IU10)”, and “nervousness (GA1)→frustration (IU2)” (see Table S2). Notably, we observed cyclic feedback between “nervousness (GA1)” and “frustration (IU2)”, while the predictive relationship from “nervousness (GA1)” to “frustration (IU2)” was stronger than the vice versa.

**Fig. 2:**
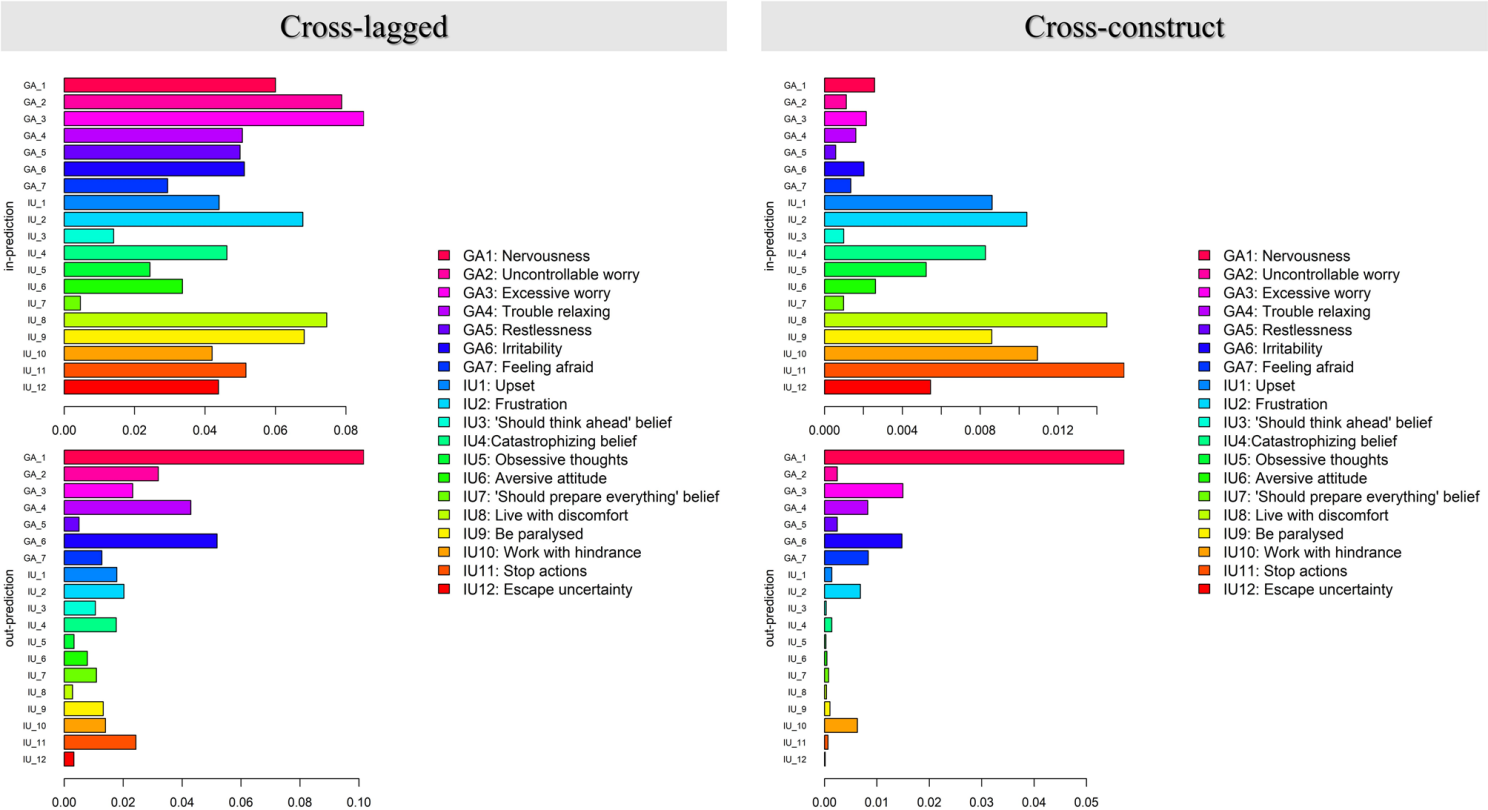
In-prediction and out-prediction for the cross-lagged and cross-construct of the CLPN

Figure 2 displays the in-prediction and out-prediction estimates of cross-lagged and cross-construct analyses. The cross-lagged results showed that “nervousness (GA1)” appeared to exert the most influence on all other nodes due to its highest out-prediction value, while “excessive worry (GA3)” seemed to be the most influenced due to its highest in-prediction value. However, due to the strong links between IU and generalized anxiety in the network, it was difficult to distinguish the reciprocal influences between these two constructs. Consequently, cross-construct analysis emerged as a particularly informative approach. As shown in Table 4, both “frustration (IU2)” and “work with hindrance (IU10)” demonstrated the strongest predictive effects on future generalized anxiety symptoms, with nearly equivalent cross-construct out-prediction values. Meanwhile, “nervousness (GA1)” emerged as the most influential generalized anxiety symptom in predicting future IU elements, given its highest cross-construct out-prediction value. Notably, generalized anxiety symptoms appeared to be more influential than IU elements overall, as most of generalized anxiety symptoms showed higher cross-construct out-prediction values than IU elements (see Table 4).

**Table 4:** The cross-lagged and cross-construct out-prediction and in-prediction for IU elements and generalized anxiety symptoms in the CLPN

Node	Cross-lagged out-prediction	Cross-lagged in-prediction	Cross-construct out-prediction	Cross-construct in-prediction
GA1	0.102	0.060	0.057	0.003
GA2	0.032	0.079	0.002	0.001
GA3	0.023	0.085	0.015	0.002
GA4	0.043	0.051	0.008	0.002
GA5	0.005	0.050	0.002	0.001
GA6	0.052	0.051	0.015	0.002
GA7	0.013	0.029	0.008	0.001
IU1	0.018	0.044	0.001	0.009
IU2	0.020	0.068	0.007	0.010
IU3	0.011	0.014	0.000	0.001
IU4	0.018	0.046	0.001	0.008
IU5	0.003	0.024	0.000	0.005
IU6	0.008	0.034	0.000	0.003
IU7	0.011	0.005	0.001	0.001
IU8	0.003	0.075	0.000	0.015
IU9	0.013	0.068	0.001	0.009
IU10	0.014	0.042	0.006	0.011
IU11	0.024	0.052	0.001	0.015
IU12	0.003	0.044	0.000	0.005

*GA* generalized anxiety,* IU* intolerance of uncertainty

### Network stability and accuracy

Based on the case-dropping test results (see Figure S4), the stability of the network was moderate (CS-C _edge_ = 0.61, CS-C _in−EI_ = 0.75, and CS-C _out−EI_ = 0.55). Edge weight difference tests and centrality difference tests were also presented (see Figures S6-S7). Additionally, the 95% bootstrapped CIs of the edges were small to moderate (see Figure S5), suggesting that the accuracy of the network was acceptable.

### Sensitivity analyses

To explore whether the relationships between IU elements and generalized anxiety symptoms vary by the level of generalized anxiety symptoms, we conducted a sensitivity analysis by dividing the sample into two groups based on the clinical cutoff score of the GAD-7: adolescents with scores ≥ 10 were classified as the high-anxiety group (*n* = 596), and those with scores < 10 as the low-anxiety group (*n* = 6,838). The edge weights are presented in Tables S3-S4, and autoregressive coefficients in Figure S12. Network structures - with and without autoregressive edges - are shown in Figures S8 and S9, using the same edge threshold (0.014) to retain only meaningful edges. Figures S10-S11 display the in-prediction and out-prediction values for cross-lagged and cross-construct analyses, while Figures S13-S14 show network stability and accuracy. Difference tests on edge weights and node centrality are reported in Figures S15-S16. The results revealed notable differences between the two groups. Specifically, the low-anxiety group exhibited a more densely connected network, with generalized anxiety symptoms exerting stronger predictive effects on IU elements. Conversely, the high-anxiety group displayed a relatively sparser network, in which IU elements had greater predictive influence on generalized anxiety symptoms. These findings suggest that the relationship between IU elements and generalized anxiety symptoms may not be static but instead shifts as a function of generalized anxiety severity.

## Discussion

Although IU has been recently recognized as a key vulnerability factor that predisposes adolescents to developing generalized anxiety [7], research exploring the temporal and causal relationships between IU and generalized anxiety in adolescents remains limited. The predominant focus on IU’s influence on generalized anxiety overlooks the potential reciprocal relationship. Using a CLPN approach, this study examined how IU elements and generalized anxiety symptoms interact over time in adolescents. Our findings revealed the dynamic nature of these relationships, providing new insights into the reciprocal interplay between IU and generalized anxiety. These findings underscore the need for interventions targeting the reciprocal relationship between IU and generalized anxiety.

### Bidirectional relationships between IU and generalized anxiety

This study revealed complex bidirectional predictive relationships between IU elements and generalized anxiety symptoms, suggesting a cyclical interaction where these constructs reinforce each other over time. This finding was consistent with earlier observations of bidirectional predictive relationships between IU and worry in adolescents [24], which extended the existing viewpoint that IU is a risk factor [21, 22] and a necessary condition [65] for adolescent generalized anxiety, advancing our understanding of the well-documented association between IU and generalized anxiety during adolescence [7]. Besides, by identifying this cyclical dynamic between IU elements and generalized anxiety symptoms through a longitudinal lens, the present study also provides evidence for the potential feedback effects between vulnerabilities and mental health problems in adolescents [63]. This self-reinforcing cycle suggests that generalized anxiety functions both as a consequence of IU and as a catalyst for its exacerbation. Effective interventions should adopt a holistic perspective, targeting both the mental disorder and its underlying risk factors simultaneously to break this cycle.

### Relatively stronger predictive relationships from generalized anxiety to IU

Further analysis revealed that generalized anxiety appeared to have a stronger predictive influence on IU, as many of the most robust pathways in the directed network were from generalized anxiety symptoms to IU elements. Additionally, IU elements demonstrated strong cross-construct in-prediction, indicating that generalized anxiety played a significant role in shaping IU. This finding is unexpected, as it challenges the conventional view that IU precedes and contributes to generalized anxiety [7]. Yet, evidence from previous related research supports the possibility of feedback effects, where mental disorders influence their associated vulnerabilities. For instance, depressive symptoms, which frequently co-occur with generalized anxiety symptoms, have been shown to predict changes in cognitive emotion regulation strategies [56] and the development of negative cognitive styles in adolescents [63], which are traditionally regarded as the risk factors of depressive symptoms. Thus, along a similar vein, adolescents may possibly experience a “scar-like” process for generalized anxiety symptoms: that is, cognitive, emotional, behavioral, or even biological changes following elevated generalized anxiety symptoms may lead to a stable increase in vulnerability, thereby increasing the likelihood of generalized anxiety recurrence in the future. Further longitudinal and clinical studies are warranted to investigate this potential process and uncover its mechanisms, which may inform targeted interventions aimed at preventing the development of chronic vulnerability and interrupting the cycle of recurrent generalized anxiety.

### Key symptoms and elements in the longitudinal interplay of IU and generalized anxiety

In the CLPN, we found that the relationships from “nervousness (GA1)” to “live with discomfort (IU8)”, from “nervousness (GA1)” to “work with hindrance (IU10)”, and from “nervousness (GA1)” to “frustration (IU2)” were the top three strongest pathways. In line with these, “nervousness (GA1)” exhibited the highest cross-lagged and cross-construct out-prediction, while “live with discomfort (IU8),” “work with hindrance (IU10),” and “frustration (IU2)” demonstrated high cross-construct in-prediction. According to prior research, “nervousness (GA1)” is considered a core symptom of generalized anxiety in the DSM-5 [66] and the most effective treatment target for reducing generalized anxiety severity during adolescence [67]. Similarly, “live with discomfort (IU8)” and “work with hindrance (IU10)” are key elements linking various IU elements, while “frustration (IU2)” acts as a central driver of IU in adolescents [34]. Thus, in addition to their central roles in respective constructs, the current findings suggest that these symptoms and elements also play pivotal roles in the longitudinal interactions between IU and generalized anxiety.

Interestingly, while “frustration (IU2)” and “work with hindrance (IU10)” were strongly influenced by generalized anxiety symptoms, they also demonstrated the highest ability to predict future generalized anxiety symptoms due to their strong cross-construct out-prediction. In addition, “excessive worry (GA3)” showed strong predictive power for IU (the second-highest in cross-construct out-prediction) but was strongly impacted by other generalized anxiety symptoms (high cross-lagged in-prediction but low cross-construct in-prediction). These findings provide important insights into the potential mechanisms underlying the bidirectional influences between IU and generalized anxiety. Specifically, “nervousness (GA1)” may serve as an initially key driver, activating both IU elements and other generalized anxiety symptoms, while “excessive worry (GA3)” - the symptom easily impacted by other generalized anxiety symptoms - may join with “nervousness (GA1)” in triggering IU elements, particularly “live with discomfort (IU8),” “work with hindrance (IU10),” and “frustration (IU2).” Among the three most susceptible IU elements, “work with hindrance (IU10)” and “frustration (IU2)” may further activate more generalized anxiety symptoms, creating a self-reinforcing vicious cycle. Thus, “work with hindrance (IU10)” and “frustration (IU2)” may serve as sensitive early indicators of IU’s interaction with generalized anxiety due to their pronounced vulnerability to generalized anxiety influence. Meanwhile, “excessive worry (GA3)” may be a critical early intervention target for reducing the risk of IU development, alongside “nervousness (GA1).”

### Potential influence of generalized anxiety severity

The sensitivity analysis revealed an intriguing shift in the directionality of associations between IU elements and generalized anxiety symptoms across levels of generalized anxiety symptoms. Among low-anxiety adolescents, generalized anxiety symptoms appeared to have a greater influence on IU elements, potentially reflecting a dynamic vulnerability process in which transient emotional distress heightens sensitivity to uncertainty. In contrast, in the high-anxiety group, IU elements played a more dominant predictive role, suggesting that in more severe generalized anxious states, IU may become a maintenance mechanism that perpetuates generalized anxiety. This pattern may reflect a developmental progression in which IU transitions from a reactive vulnerability to a more entrenched cognitive-emotional style in adolescents with elevated anxiety. However, given the relatively small sample size and low network stability in the high-anxiety group, these findings should be interpreted with caution. Future research in larger clinical samples with a broader range of generalized anxiety severity is needed to further clarify the temporal dynamics between IU elements and generalized anxiety symptoms across different levels of generalized anxiety symptoms.

### Implications

This study offers several practical implications. First, the bidirectional predictive relationships between generalized anxiety and IU highlight the importance of addressing both constructs simultaneously in clinical and therapeutic settings. Interventions targeting one may inadvertently influence the other, underscoring the need for integrated approaches. Furthermore, the relatively stronger predictive effect of generalized anxiety on IU challenges traditional notions and suggests that early intervention for generalized anxiety could help mitigate its associated vulnerabilities, such as IU. Second, the strong predictive power of “nervousness (GA1)” for subsequent generalized anxiety symptoms and IU elements emphasizes the importance of identifying and managing adolescents’ nervous emotions early. Teaching effective strategies, such as relaxation techniques, could prevent the escalation of generalized anxiety symptoms and IU elements. Third, the key roles of “work with hindrance (IU10)” and “frustration (IU2)” (both heavily influenced by generalized anxiety symptoms and capable of activating other generalized anxiety symptoms) suggest that targeting these emotional and behavioral restrictions may be particularly effective in disrupting the cyclical relationship between IU and generalized anxiety. Intervention programs, such as IU-based cognitive behavioral therapy (CBT-IU) for generalized anxiety [23], could benefit from incorporating instructions on emotion regulation strategies and practical training on managing everyday uncertainties.

### Limitations

This study has several limitations. First, all variables were measured through self-report questionnaires, which may introduce bias. Future research should include a variety of assessment methods, such as objective measures and clinical diagnoses, to validate and expand upon these findings. Second, although this study used a cross-lagged network to examine interactions between IU and generalized anxiety, caution is warranted when interpreting causality. The six-month time interval between waves may have missed more detailed dynamics within shorter periods. Future research should adopt more granular longitudinal designs, such as time-series network analyses, with additional follow-ups and longer observation periods to capture nuanced changes in the relationships between IU and generalized anxiety. Third, this study focused exclusively on nonclinical adolescents, which may limit the generalizability of findings to clinical populations. However, studying nonclinical samples is critical for understanding how IU influences generalized anxiety in the absence of medication or institutional effects. Future research should extend these findings by including clinically relevant samples of adolescents to evaluate the broader applicability of the results. Fourth, in light of the central role of stressful events in the Uncertainty Distress Model [68], we incorporated this variable as a covariate in our CLPN analyses to control for its potential confounding effects. Future research could conduct subgroup analyses by stratifying adolescents based on the intensity or frequency of stressful events to further explore how the associations between IU elements and generalized anxiety symptoms may vary across stress exposure levels. Finally, the current sample was drawn from a single metropolitan city in China, and as such, cultural and contextual factors may have influenced our findings. For example, Chinese collectivist culture places a strong emphasis on social and family relationships, which may affect how adolescents experience and cope with anxiety and uncertainty. This cultural context could lead adolescents to prioritize family and societal expectations, and such pressures may, in turn, impact their levels of generalized anxiety and IU. Additionally, adolescents living in urban environments often face unique stressors, such as academic pressure, social competition, and concerns about their future, which may differ from the challenges faced by those in rural areas. These urban-specific factors may have influenced the present findings in ways that may not necessarily apply to adolescents from smaller towns or rural regions, where socioeconomic pressures and access to mental health resources can vary significantly. Future research should replicate the current findings in more diverse populations, including adolescents from different cultural and contextual settings, to enhance the generalizability and applicability of these findings.

## Conclusions

From a network perspective, this study extended existing research by disentangling the bidirectional predictive relationships between IU and generalized anxiety among adolescents. These findings revealed that IU not only drives the development of generalized anxiety but is also exacerbated by it, offering a nuanced temporal perspective on their cyclical interaction. Additionally, the identification of key IU elements and generalized anxiety symptoms in their longitudinal interactions informs potential intervention targets for breaking the vicious cycle of IU and generalized anxiety, offering a pathway to more effective prevention and treatment strategies for adolescents. Future research should further investigate the dynamic interplay between IU and generalized anxiety to deepen our understanding of the mechanisms underlying their relationship. These efforts will be instrumental in refining therapeutic approaches and improving outcomes for adolescents struggling with generalized anxiety and IU.

## Electronic supplementary material

Below is the link to the electronic supplementary material.

Supplementary Material 1.

## Data Availability

Please contact PhD Fang Fan at fangfan@scnu.edu.cn for data supporting the findings of the current study.
