# Supplementary material for: Bidirectional relationships between intolerance of uncertainty and generalized anxiety among adolescents: insights from cross-lagged panel network analysis
Source: Child Adolesc Psychiatry Ment Health. 2025 May 14;19:54. doi: 10.1186/s13034-025-00912-6 (PMC12079871; doi:10.1186/s13034-025-00912-6)
Supplement: Supplementary file 1 — Supplementary Material 1. [file 13034_2025_912_MOESM1_ESM.docx]

**Supplementary materials**

**Detailed network analysis procedure**

***Network estimation***

The cross-lagged panel network (CLPN) was estimated to explore the interrelationships between IU components and generalized anxiety symptoms via the R-package “glmnet” (Friedman et al., 2010). The CLPN can depict how nodes at the first time point predict nodes at the second time point after adjusting all other variables at the first time point, whereas other longitudinal network estimation methods require data from at least three time points (Epskamp, 2020). We applied the LASSO method to shrink the small regression path to 0 and chose the best-penalized model with a 10-fold cross-validation method. Each edge in the CLPN signifies a directed regression path: the edges of each node pointing to itself signify the autoregression coefficients, and the edges pointing to other nodes signify the cross-lagged regression coefficients. The thickness and color depth of the edge represent the strength of this regression path. The blue and red arrows indicate positive and negative effects, respectively.

***Centrality calculation***

We estimated two centrality indices - out-prediction and in-prediction - through the R-package “lavaan” (Rosseel, 2012). The in-prediction is the extent to which each node is influenced by other nodes in the network, and out-prediction is the extent to which each node predicts other variables in the network. The higher the in-prediction, the more it is influenced by other nodes, while the higher the out-prediction, the more it influences the other nodes. In line with previous CLPN studies on risk factors and mental disorders (Bernstein et al., 2019; Sun et al., 2024; Yuan et al., 2024), we calculated the in-prediction and out-prediction for cross-lagged (excluding autoregressive path of the node of interest) and cross-construct (excluding autoregressive path and paths within the same construct). The cross-lagged analysis examines the proportion of variance accounted for by all other variables at the previous measurement time, while the cross-construct in-prediction examines the proportion of variance accounted for by all variables that belong to a different construct at the previous measurement time.

***Network accuracy and stability***

First, a total of 5000 nonparametric bootstraps were used to assess the 95% confidence intervals (CIs) of edge weights (Epskamp et al., 2018). The narrower CIs indicated a more accurate network. Additionally, we estimated the 95% bootstrapped (1000 iterations) CIs of differences between edge weights and centrality indices to test whether these differences were statistically significant.

Second, a total of 5000 case-drop bootstraps were used to estimate the stability of edge-weights and centrality indices (Epskamp et al., 2018), which can provide the correlation stability coefficient (CS-C), a measure that indicates the maximal proportion of cases that can be dropped to maintain a correlation higher than 0.70 between the centrality indexes computed via the original network and those via the new network estimated without the dropped cases. Generally, CS-C should be ≥ 0.25, preferably ≥ 0.5 (Rhemtulla et al., 2022).

**References**

Bernstein, E.E., Kleiman, E.M., van Bork, R., Moriarity, D.P., Mac Giollabhui, N., McNally, R.J., Abramson, L.Y., Alloy, L.B. (2019). Unique and predictive relationships between components of cognitive vulnerability and symptoms of depression. Depression and Anxiety, 36(10), 950-959. https://doi.org/10.1002/da.22935

Epskamp, S. (2020). Psychometric network models from time-series and panel data. Psychometrika, 85(1), 206-231. https://doi.org/10.1007/s11336-020-09697-3

Epskamp, S., Borsboom, D., Fried, E.I. (2018). Estimating psychological networks and their accuracy: A tutorial paper. Behavior Research Methods, 50(1), 195-212. https://doi.org/10.3758/s13428-017-0862-1

Friedman, J., Hastie, T., Tibshirani, R. (2010). Regularization Paths for Generalized Linear Models via Coordinate Descent. Journal of Statistical Software, 33(1), 1-22.

Rhemtulla, M., van Bork, R., Cramer, A. (2022). Cross-lagged network models. Multivariate Behavioral Research,

Rosseel, Y. (2012). lavaan: An R Package for Structural Equation Modeling. Journal of Statistical Software, 48(2), 1-36. https://doi.org/10.18637/jss.v048.i02

Sun, X., Yuan, T., Chen, F., Li, Y., Jiang, N. (2024). Network analysis of maternal parenting practices and adolescent mental health problems: a longitudinal study. Child and Adolescent Psychiatry and Mental Health, 18(1), 38. https://doi.org/10.1186/s13034-024-00728-w

Yuan, T., Sun, J., Wang, X., Sun, X., Chen, F., Li, C., Li, Y., Jiang, N. (2024). Unique longitudinal relationships between depressive symptoms and cognitive emotion regulation strategies in Chinese adolescents: a cross-lagged panel network analysis. Current Psychology, 43(46), 35364-35376. https://doi.org/10.1007/s12144-024-06981-8

**Table S1.** LASSO cross-lagged regression matrix of IU elements and generalized anxiety symptoms.

|  | GA_1 | GA_2 | GA_3 | GA_4 | GA_5 | GA_6 | GA_7 | IU_1 | IU_2 | IU_3 | IU_4 | IU_5 | IU_6 | IU_7 | IU_8 | IU_9 | IU_10 | IU_11 | IU_12 |
| --- | --- | --- | --- | --- | --- | --- | --- | --- | --- | --- | --- | --- | --- | --- | --- | --- | --- | --- | --- |
| GA_1 | 0.14 | 0.04 | 0.07 | 0.06 | 0.06 | 0.07 | 0.04 | 0.04 | 0.05 | 0.02 | 0.05 | 0.03 | 0.03 | 0.03 | 0.06 | 0.03 | 0.06 | 0.05 | 0.02 |
| GA_2 | 0.05 | 0.10 | 0.06 | 0.06 | 0.05 | 0.06 | 0.03 | 0.01 | 0.01 | 0.00 | 0.00 | 0.00 | 0.00 | 0.00 | 0.01 | 0.00 | 0.00 | 0.00 | 0.00 |
| GA_3 | 0.01 | 0.05 | 0.09 | 0.00 | 0.02 | 0.00 | 0.02 | 0.01 | 0.00 | 0.01 | 0.00 | 0.01 | 0.00 | 0.00 | 0.01 | 0.01 | 0.03 | 0.04 | 0.02 |
| GA_4 | 0.10 | 0.07 | 0.09 | 0.15 | 0.06 | 0.07 | 0.03 | 0.03 | 0.01 | 0.00 | 0.03 | 0.00 | 0.02 | 0.00 | 0.02 | 0.04 | 0.00 | 0.01 | 0.00 |
| GA_5 | 0.00 | 0.03 | 0.00 | 0.01 | 0.12 | 0.00 | 0.04 | 0.00 | 0.00 | 0.00 | -0.01 | 0.00 | 0.00 | 0.00 | 0.02 | 0.02 | 0.00 | 0.00 | 0.01 |
| GA_6 | 0.06 | 0.06 | 0.04 | 0.07 | 0.03 | 0.15 | 0.00 | 0.00 | 0.02 | 0.00 | 0.01 | 0.00 | 0.00 | 0.00 | 0.00 | 0.00 | 0.02 | 0.03 | 0.01 |
| GA_7 | 0.00 | 0.03 | 0.02 | 0.00 | 0.02 | 0.00 | 0.17 | 0.00 | 0.01 | 0.00 | 0.02 | 0.03 | 0.00 | 0.00 | 0.02 | 0.00 | 0.00 | 0.01 | 0.03 |
| IU_1 | 0.01 | 0.01 | 0.00 | 0.00 | 0.00 | 0.02 | 0.00 | 0.16 | 0.05 | 0.00 | 0.03 | 0.02 | 0.06 | 0.00 | 0.05 | 0.02 | 0.03 | 0.05 | 0.03 |
| IU_2 | 0.02 | 0.01 | 0.02 | 0.01 | 0.01 | 0.01 | 0.02 | 0.02 | 0.10 | 0.00 | 0.00 | 0.04 | 0.01 | 0.00 | 0.04 | 0.03 | 0.00 | 0.00 | 0.04 |
| IU_3 | 0.00 | 0.00 | 0.00 | 0.00 | 0.00 | -0.01 | -0.01 | -0.03 | -0.02 | 0.14 | -0.02 | -0.01 | 0.00 | 0.06 | -0.02 | -0.01 | -0.01 | -0.04 | -0.01 |
| IU_4 | 0.01 | 0.01 | 0.01 | 0.00 | 0.00 | 0.00 | 0.00 | 0.06 | 0.04 | 0.00 | 0.15 | 0.00 | 0.04 | 0.00 | 0.01 | 0.04 | 0.03 | 0.05 | 0.02 |
| IU_5 | 0.00 | 0.00 | 0.00 | 0.00 | -0.02 | 0.00 | 0.00 | 0.02 | 0.00 | 0.01 | 0.00 | 0.21 | 0.03 | 0.00 | 0.00 | 0.00 | 0.00 | 0.00 | 0.00 |
| IU_6 | 0.00 | 0.01 | 0.00 | 0.00 | -0.01 | 0.00 | 0.00 | 0.03 | 0.01 | 0.02 | 0.06 | 0.02 | 0.16 | 0.00 | 0.00 | 0.00 | 0.03 | 0.01 | 0.00 |
| IU_7 | 0.00 | -0.02 | 0.00 | 0.00 | 0.00 | 0.00 | 0.00 | 0.00 | -0.02 | 0.08 | 0.00 | -0.01 | 0.00 | 0.16 | -0.02 | -0.03 | 0.00 | -0.01 | -0.01 |
| IU_8 | 0.00 | 0.00 | 0.00 | 0.02 | 0.01 | 0.00 | 0.00 | 0.00 | 0.02 | 0.00 | 0.00 | 0.00 | 0.00 | -0.02 | 0.08 | 0.04 | 0.00 | 0.00 | 0.00 |
| IU_9 | 0.00 | 0.00 | 0.00 | 0.00 | 0.01 | 0.00 | 0.00 | 0.01 | 0.05 | 0.00 | 0.00 | 0.00 | 0.00 | 0.00 | 0.06 | 0.09 | 0.01 | 0.02 | 0.06 |
| IU_10 | 0.01 | 0.00 | 0.02 | 0.01 | 0.00 | 0.01 | 0.01 | 0.01 | 0.00 | 0.00 | 0.02 | 0.00 | 0.01 | 0.00 | 0.00 | 0.02 | 0.15 | 0.03 | 0.01 |
| IU_11 | 0.00 | 0.00 | 0.00 | 0.00 | 0.01 | 0.01 | 0.00 | 0.03 | 0.06 | 0.00 | 0.07 | 0.04 | 0.02 | 0.00 | 0.04 | 0.06 | 0.05 | 0.15 | 0.05 |
| IU_12 | 0.00 | 0.00 | 0.00 | 0.00 | 0.00 | 0.01 | 0.02 | 0.01 | 0.01 | 0.00 | 0.00 | -0.01 | 0.00 | 0.00 | 0.03 | 0.04 | 0.00 | 0.03 | 0.13 |

**Note:**

GA: generalized anxiety; IU: intolerance of uncertainty.

Independent variables (i.e., predictors) are in rows, and dependent variables are in columns. Autoregressive edges are presented along the diagonal. Each number in the matrix represents the regression coefficient of the node in the same row on its left side (measured at T1) predicting the node in the same column on its upper side (measured at T2). All models covaried for multiple sociodemographics.

**Table S2** The directed edge weights between IU components and generalized anxiety symptoms in the CLPN.

| **NodeOut** | **NodeIn** | **Value** | **NodeOut** | **NodeIn** | **Value** |
| --- | --- | --- | --- | --- | --- |
| **GA → IU** | | | **IU → GA** | | |
| GA_1 | IU_8 | 0.0571 | IU_2 | GA_1 | 0.0247 |
| GA_1 | IU_10 | 0.0561 | IU_8 | GA_4 | 0.0216 |
| GA_1 | IU_2 | 0.0548 | IU_1 | GA_6 | 0.0203 |
| GA_1 | IU_4 | 0.0494 | IU_12 | GA_7 | 0.0195 |
| GA_1 | IU_11 | 0.0473 | IU_2 | GA_3 | 0.0183 |
| GA_4 | IU_9 | 0.0397 | IU_10 | GA_3 | 0.0172 |
| GA_1 | IU_1 | 0.0392 | IU_2 | GA_7 | 0.0152 |
| GA_3 | IU_11 | 0.0350 | IU_9 | GA_5 | 0.0145 |
| GA_3 | IU_10 | 0.0348 | IU_2 | GA_2 | 0.0140 |
| GA_4 | IU_1 | 0.0342 | IU_11 | GA_6 | 0.0136 |
| GA_7 | IU_5 | 0.0342 | IU_1 | GA_2 | 0.0134 |
| GA_1 | IU_9 | 0.0333 | IU_10 | GA_4 | 0.0123 |
| GA_1 | IU_5 | 0.0332 | IU_4 | GA_3 | 0.0121 |
| GA_7 | IU_12 | 0.0304 | IU_2 | GA_6 | 0.0119 |
| GA_6 | IU_11 | 0.0297 | IU_10 | GA_7 | 0.0115 |
| GA_1 | IU_6 | 0.0296 | IU_10 | GA_1 | 0.0113 |
| GA_4 | IU_4 | 0.0285 | IU_4 | GA_2 | 0.0108 |
| GA_1 | IU_7 | 0.0279 | IU_4 | GA_1 | 0.0105 |
| GA_4 | IU_6 | 0.0227 | IU_2 | GA_4 | 0.0086 |
| GA_6 | IU_10 | 0.0225 | IU_2 | GA_5 | 0.0081 |
| GA_4 | IU_8 | 0.0220 | IU_12 | GA_6 | 0.0079 |
| GA_1 | IU_12 | 0.0220 | IU_10 | GA_6 | 0.0073 |
| GA_6 | IU_2 | 0.0211 | IU_6 | GA_2 | 0.0064 |
| GA_7 | IU_8 | 0.0208 | IU_1 | GA_1 | 0.0063 |
| GA_5 | IU_8 | 0.0205 | IU_11 | GA_5 | 0.0061 |
| GA_5 | IU_9 | 0.0203 | IU_8 | GA_5 | 0.0060 |
| GA_7 | IU_4 | 0.0186 | IU_11 | GA_3 | 0.0045 |
| GA_1 | IU_3 | 0.0183 | IU_6 | GA_1 | 0.0043 |
| GA_3 | IU_12 | 0.0179 | IU_12 | GA_5 | 0.0043 |
| GA_2 | IU_2 | 0.0148 | IU_1 | GA_4 | 0.0024 |
| GA_7 | IU_11 | 0.0145 | IU_4 | GA_7 | 0.0020 |
| GA_3 | IU_1 | 0.0143 | IU_8 | GA_7 | 0.0013 |
| GA_6 | IU_4 | 0.0134 | IU_11 | GA_2 | 0.0011 |
| GA_3 | IU_3 | 0.0132 | IU_7 | GA_7 | -0.0018 |
| GA_3 | IU_5 | 0.0129 | IU_7 | GA_6 | -0.0042 |
| GA_4 | IU_11 | 0.0116 | IU_6 | GA_5 | -0.0056 |
| GA_7 | IU_2 | 0.0111 | IU_3 | GA_6 | -0.0104 |
| GA_3 | IU_9 | 0.0084 | IU_3 | GA_7 | -0.0108 |
| GA_2 | IU_8 | 0.0079 | IU_7 | GA_2 | -0.0178 |
| GA_6 | IU_12 | 0.0074 | IU_5 | GA_5 | -0.0179 |
| GA_4 | IU_2 | 0.0068 |  |  |  |
| GA_3 | IU_8 | 0.0068 |  |  |  |
| GA_2 | IU_1 | 0.0068 |  |  |  |
| GA_5 | IU_12 | 0.0063 |  |  |  |
| GA_7 | IU_1 | 0.0048 |  |  |  |
| GA_4 | IU_7 | 0.0047 |  |  |  |
| GA_3 | IU_4 | 0.0041 |  |  |  |
| GA_3 | IU_2 | 0.0039 |  |  |  |
| GA_6 | IU_6 | 0.0034 |  |  |  |
| GA_6 | IU_1 | 0.0033 |  |  |  |
| GA_4 | IU_3 | 0.0026 |  |  |  |
| GA_7 | IU_9 | 0.0010 |  |  |  |
| GA_6 | IU_7 | -0.0002 |  |  |  |
| GA_5 | IU_4 | -0.0148 |  |  |  |

**Note:**

GA: generalized anxiety; IU: intolerance of uncertainty.

**Table S3.** LASSO cross-lagged regression matrix of IU elements and generalized anxiety symptoms for the low-anxiety group.

|  | GA_1 | GA_2 | GA_3 | GA_4 | GA_5 | GA_6 | GA_7 | IU_1 | IU_2 | IU_3 | IU_4 | IU_5 | IU_6 | IU_7 | IU_8 | IU_9 | IU_10 | IU_11 | IU_12 |
| --- | --- | --- | --- | --- | --- | --- | --- | --- | --- | --- | --- | --- | --- | --- | --- | --- | --- | --- | --- |
| GA_1 | 0.13 | 0.04 | 0.06 | 0.06 | 0.05 | 0.08 | 0.04 | 0.04 | 0.05 | 0.01 | 0.05 | 0.03 | 0.02 | 0.03 | 0.05 | 0.03 | 0.05 | 0.05 | 0.02 |
| GA_2 | 0.05 | 0.08 | 0.06 | 0.06 | 0.05 | 0.05 | 0.04 | 0.01 | 0.02 | 0.00 | 0.01 | 0.00 | 0.00 | 0.00 | 0.01 | 0.00 | 0.00 | 0.00 | 0.00 |
| GA_3 | 0.01 | 0.04 | 0.08 | 0.00 | 0.01 | 0.00 | 0.01 | 0.00 | 0.00 | 0.02 | 0.00 | 0.01 | 0.00 | 0.00 | 0.01 | 0.00 | 0.02 | 0.03 | 0.01 |
| GA_4 | 0.08 | 0.06 | 0.07 | 0.13 | 0.05 | 0.07 | 0.03 | 0.03 | 0.02 | 0.01 | 0.04 | 0.00 | 0.02 | 0.00 | 0.02 | 0.04 | 0.01 | 0.03 | 0.00 |
| GA_5 | 0.00 | 0.03 | 0.00 | 0.01 | 0.09 | 0.00 | 0.03 | 0.00 | 0.00 | -0.01 | 0.00 | 0.00 | 0.00 | 0.00 | 0.01 | 0.02 | 0.00 | 0.00 | 0.00 |
| GA_6 | 0.06 | 0.06 | 0.04 | 0.06 | 0.02 | 0.14 | 0.00 | 0.00 | 0.01 | 0.00 | 0.00 | 0.00 | 0.00 | -0.01 | 0.00 | 0.00 | 0.00 | 0.01 | 0.00 |
| GA_7 | 0.00 | 0.03 | 0.03 | 0.01 | 0.02 | 0.00 | 0.15 | 0.00 | 0.00 | 0.00 | 0.02 | 0.02 | 0.00 | 0.00 | 0.01 | 0.00 | 0.00 | 0.02 | 0.02 |
| IU_1 | 0.01 | 0.02 | 0.01 | 0.00 | 0.00 | 0.03 | 0.00 | 0.16 | 0.05 | 0.00 | 0.03 | 0.01 | 0.06 | 0.00 | 0.05 | 0.02 | 0.03 | 0.05 | 0.04 |
| IU_2 | 0.03 | 0.01 | 0.02 | 0.01 | 0.00 | 0.02 | 0.00 | 0.02 | 0.09 | -0.02 | 0.00 | 0.03 | 0.03 | 0.00 | 0.03 | 0.01 | 0.00 | 0.00 | 0.02 |
| IU_3 | 0.00 | 0.00 | 0.00 | 0.00 | 0.00 | -0.01 | 0.00 | -0.02 | 0.00 | 0.14 | 0.00 | 0.00 | 0.00 | 0.06 | -0.01 | -0.01 | 0.00 | -0.03 | -0.02 |
| IU_4 | 0.00 | 0.00 | 0.00 | 0.00 | 0.00 | 0.00 | 0.00 | 0.05 | 0.03 | 0.01 | 0.14 | 0.00 | 0.03 | 0.00 | 0.01 | 0.04 | 0.01 | 0.04 | 0.01 |
| IU_5 | 0.00 | 0.00 | 0.00 | 0.00 | 0.00 | 0.00 | 0.00 | 0.01 | 0.00 | 0.01 | 0.00 | 0.20 | 0.03 | 0.00 | 0.00 | 0.00 | 0.00 | 0.00 | 0.00 |
| IU_6 | 0.01 | 0.01 | 0.01 | 0.00 | 0.00 | 0.00 | 0.00 | 0.03 | 0.00 | 0.04 | 0.05 | 0.01 | 0.15 | 0.00 | 0.00 | 0.00 | 0.02 | 0.00 | 0.00 |
| IU_7 | 0.00 | -0.02 | 0.00 | 0.00 | 0.00 | -0.01 | 0.00 | 0.00 | 0.00 | 0.08 | 0.00 | 0.00 | 0.00 | 0.16 | -0.02 | -0.03 | 0.00 | -0.02 | -0.02 |
| IU_8 | 0.00 | 0.00 | 0.00 | 0.02 | 0.00 | 0.00 | 0.00 | 0.00 | 0.01 | -0.01 | 0.00 | 0.00 | 0.00 | -0.03 | 0.07 | 0.05 | 0.00 | 0.01 | 0.01 |
| IU_9 | 0.00 | 0.00 | 0.00 | 0.00 | 0.01 | 0.00 | 0.00 | 0.02 | 0.05 | 0.00 | 0.00 | 0.02 | 0.00 | 0.00 | 0.09 | 0.10 | 0.02 | 0.03 | 0.07 |
| IU_10 | 0.01 | 0.00 | 0.01 | 0.01 | 0.00 | 0.01 | 0.01 | 0.01 | 0.00 | -0.01 | 0.02 | 0.00 | 0.01 | 0.00 | 0.00 | 0.02 | 0.15 | 0.04 | 0.01 |
| IU_11 | 0.00 | 0.00 | 0.00 | 0.00 | 0.00 | 0.00 | 0.00 | 0.03 | 0.06 | 0.00 | 0.06 | 0.03 | 0.00 | 0.00 | 0.03 | 0.05 | 0.04 | 0.13 | 0.04 |
| IU_12 | 0.00 | 0.00 | 0.00 | 0.00 | 0.01 | 0.01 | 0.02 | 0.00 | 0.00 | 0.00 | 0.00 | 0.00 | 0.00 | 0.00 | 0.03 | 0.04 | 0.00 | 0.03 | 0.12 |

**Note:**

GA: generalized anxiety; IU: intolerance of uncertainty.

Independent variables (i.e., predictors) are in rows, and dependent variables are in columns. Autoregressive edges are presented along the diagonal. Each number in the matrix represents the regression coefficient of the node in the same row on its left side (measured at T1) predicting the node in the same column on its upper side (measured at T2). All models covaried for multiple sociodemographics.

**Table S4.** LASSO cross-lagged regression matrix of IU elements and generalized anxiety symptoms for the high-anxiety group.

|  | GA_1 | GA_2 | GA_3 | GA_4 | GA_5 | GA_6 | GA_7 | IU_1 | IU_2 | IU_3 | IU_4 | IU_5 | IU_6 | IU_7 | IU_8 | IU_9 | IU_10 | IU_11 | IU_12 |
| --- | --- | --- | --- | --- | --- | --- | --- | --- | --- | --- | --- | --- | --- | --- | --- | --- | --- | --- | --- |
| GA_1 | 0.07 | 0.00 | 0.00 | 0.00 | 0.02 | 0.00 | 0.00 | 0.00 | 0.01 | 0.00 | 0.00 | 0.00 | 0.00 | 0.00 | 0.01 | 0.00 | 0.00 | 0.00 | 0.00 |
| GA_2 | 0.00 | 0.06 | 0.00 | 0.02 | 0.00 | 0.00 | 0.00 | 0.00 | 0.00 | -0.01 | 0.00 | 0.00 | -0.02 | 0.00 | 0.00 | 0.00 | 0.00 | 0.00 | 0.00 |
| GA_3 | 0.00 | 0.00 | 0.07 | 0.01 | 0.00 | 0.00 | 0.02 | 0.00 | 0.01 | 0.00 | 0.00 | 0.00 | 0.00 | 0.00 | 0.00 | 0.00 | 0.06 | 0.00 | 0.04 |
| GA_4 | 0.06 | 0.07 | 0.06 | 0.09 | 0.04 | 0.05 | 0.00 | 0.00 | 0.00 | 0.00 | 0.00 | 0.00 | 0.00 | 0.00 | 0.00 | 0.00 | 0.00 | 0.00 | 0.00 |
| GA_5 | 0.00 | 0.00 | 0.00 | 0.00 | 0.12 | 0.00 | 0.03 | 0.00 | 0.00 | 0.00 | 0.00 | 0.00 | 0.01 | 0.00 | 0.03 | 0.00 | 0.00 | 0.00 | 0.02 |
| GA_6 | 0.00 | 0.00 | 0.00 | 0.01 | 0.00 | 0.09 | 0.00 | 0.04 | 0.03 | 0.00 | 0.02 | 0.00 | 0.04 | 0.00 | 0.00 | 0.00 | 0.06 | 0.04 | 0.00 |
| GA_7 | 0.00 | 0.00 | 0.00 | 0.00 | -0.01 | 0.00 | 0.09 | 0.00 | 0.03 | 0.00 | 0.00 | 0.02 | 0.00 | 0.00 | 0.03 | 0.00 | -0.01 | 0.00 | 0.02 |
| IU_1 | 0.00 | 0.00 | 0.00 | 0.01 | 0.04 | 0.00 | 0.00 | 0.07 | 0.00 | 0.00 | 0.00 | 0.00 | 0.00 | 0.00 | 0.00 | 0.00 | 0.00 | 0.00 | 0.00 |
| IU_2 | 0.00 | 0.00 | 0.00 | 0.00 | 0.00 | 0.00 | 0.04 | 0.00 | 0.13 | 0.00 | 0.01 | 0.04 | 0.00 | 0.00 | 0.02 | 0.08 | 0.04 | 0.02 | 0.06 |
| IU_3 | 0.00 | 0.00 | 0.00 | 0.00 | 0.00 | 0.00 | 0.00 | 0.00 | 0.00 | 0.09 | -0.01 | 0.00 | 0.00 | 0.10 | 0.00 | 0.00 | 0.00 | -0.01 | 0.00 |
| IU_4 | 0.01 | 0.07 | 0.07 | 0.01 | 0.00 | 0.03 | 0.00 | 0.07 | 0.01 | 0.00 | 0.17 | 0.04 | 0.04 | 0.00 | 0.02 | 0.00 | 0.06 | 0.05 | 0.01 |
| IU_5 | 0.00 | 0.00 | 0.00 | 0.00 | -0.07 | 0.00 | 0.00 | 0.04 | 0.00 | 0.02 | 0.00 | 0.18 | 0.00 | 0.00 | 0.00 | 0.00 | 0.00 | 0.00 | 0.00 |
| IU_6 | 0.00 | 0.00 | 0.00 | 0.00 | 0.00 | -0.02 | 0.00 | 0.01 | 0.00 | 0.00 | 0.05 | 0.00 | 0.19 | 0.00 | 0.05 | 0.00 | 0.01 | 0.00 | 0.00 |
| IU_7 | 0.00 | 0.00 | 0.00 | 0.00 | 0.00 | 0.00 | 0.00 | 0.00 | 0.00 | 0.12 | 0.00 | 0.00 | 0.00 | 0.15 | 0.00 | 0.00 | 0.00 | 0.00 | 0.00 |
| IU_8 | 0.00 | 0.00 | 0.00 | 0.00 | 0.00 | 0.00 | 0.00 | 0.00 | 0.03 | 0.08 | 0.00 | 0.00 | 0.00 | 0.01 | 0.05 | 0.00 | 0.00 | 0.00 | 0.00 |
| IU_9 | 0.00 | 0.00 | 0.00 | 0.00 | 0.00 | 0.00 | 0.00 | 0.00 | 0.00 | -0.07 | 0.00 | 0.00 | 0.00 | 0.00 | 0.00 | 0.00 | 0.00 | 0.00 | 0.00 |
| IU_10 | 0.00 | 0.01 | 0.04 | 0.05 | 0.00 | 0.00 | 0.00 | 0.00 | 0.00 | 0.01 | 0.00 | 0.00 | 0.00 | 0.00 | 0.00 | 0.01 | 0.03 | 0.00 | 0.00 |
| IU_11 | 0.06 | 0.05 | 0.06 | 0.02 | 0.09 | 0.07 | 0.04 | 0.04 | 0.05 | 0.00 | 0.06 | 0.00 | 0.06 | 0.01 | 0.07 | 0.11 | 0.07 | 0.18 | 0.06 |
| IU_12 | 0.00 | 0.00 | 0.00 | 0.00 | 0.00 | 0.00 | 0.00 | 0.00 | 0.03 | 0.00 | 0.00 | 0.00 | 0.00 | 0.00 | 0.00 | 0.00 | 0.00 | 0.00 | 0.12 |

**Note:**

GA: generalized anxiety; IU: intolerance of uncertainty.

Independent variables (i.e., predictors) are in rows, and dependent variables are in columns. Autoregressive edges are presented along the diagonal. Each number in the matrix represents the regression coefficient of the node in the same row on its left side (measured at T1) predicting the node in the same column on its upper side (measured at T2). All models covaried for multiple sociodemographics.

**Table S5** The directed edge weights between IU components and generalized anxiety symptoms in the CLPN of the low-anxiety group.

| **NodeOut** | **NodeIn** | **Value** | **NodeOut** | **NodeIn** | **Value** |
| --- | --- | --- | --- | --- | --- |
| **GA → IU** | | | **IU → GA** | | |
| GA_1 | IU_2 | 0.0527 | IU_2 | GA_1 | 0.0334 |
| GA_1 | IU_8 | 0.0506 | IU_1 | GA_6 | 0.0256 |
| GA_1 | IU_11 | 0.0506 | IU_1 | GA_2 | 0.0236 |
| GA_1 | IU_10 | 0.0492 | IU_8 | GA_4 | 0.0236 |
| GA_1 | IU_4 | 0.0461 | IU_12 | GA_7 | 0.0218 |
| GA_4 | IU_9 | 0.0408 | IU_2 | GA_6 | 0.0195 |
| GA_1 | IU_1 | 0.0385 | IU_2 | GA_3 | 0.0190 |
| GA_4 | IU_4 | 0.0352 | IU_2 | GA_2 | 0.0143 |
| GA_1 | IU_9 | 0.0349 | IU_6 | GA_2 | 0.0119 |
| GA_4 | IU_1 | 0.0345 | IU_10 | GA_3 | 0.0118 |
| GA_1 | IU_7 | 0.0322 | IU_12 | GA_6 | 0.0116 |
| GA_1 | IU_5 | 0.0320 | IU_9 | GA_5 | 0.0105 |
| GA_3 | IU_11 | 0.0295 | IU_10 | GA_7 | 0.0105 |
| GA_4 | IU_11 | 0.0282 | IU_1 | GA_1 | 0.0088 |
| GA_1 | IU_12 | 0.0245 | IU_10 | GA_6 | 0.0083 |
| GA_4 | IU_8 | 0.0239 | IU_10 | GA_1 | 0.0076 |
| GA_7 | IU_12 | 0.0233 | IU_10 | GA_4 | 0.0071 |
| GA_4 | IU_6 | 0.0233 | IU_6 | GA_1 | 0.0069 |
| GA_3 | IU_10 | 0.0233 | IU_6 | GA_3 | 0.0068 |
| GA_2 | IU_2 | 0.0212 | IU_2 | GA_4 | 0.0067 |
| GA_1 | IU_6 | 0.0209 | IU_1 | GA_3 | 0.0064 |
| GA_3 | IU_3 | 0.0198 | IU_12 | GA_5 | 0.0063 |
| GA_7 | IU_11 | 0.0188 | IU_8 | GA_5 | 0.0041 |
| GA_7 | IU_5 | 0.0183 | IU_4 | GA_1 | 0.0039 |
| GA_5 | IU_9 | 0.0173 | IU_8 | GA_7 | 0.0039 |
| GA_4 | IU_2 | 0.0155 | IU_2 | GA_7 | 0.0027 |
| GA_7 | IU_4 | 0.0153 | IU_1 | GA_4 | 0.0015 |
| GA_6 | IU_2 | 0.0148 | IU_3 | GA_6 | -0.0084 |
| GA_2 | IU_8 | 0.0133 | IU_7 | GA_6 | -0.0112 |
| GA_1 | IU_3 | 0.0130 | IU_7 | GA_2 | -0.0244 |
| GA_3 | IU_12 | 0.0129 |  |  |  |
| GA_2 | IU_1 | 0.0128 |  |  |  |
| GA_5 | IU_8 | 0.0126 |  |  |  |
| GA_6 | IU_11 | 0.0126 |  |  |  |
| GA_4 | IU_3 | 0.0122 |  |  |  |
| GA_2 | IU_4 | 0.0109 |  |  |  |
| GA_7 | IU_8 | 0.0088 |  |  |  |
| GA_3 | IU_5 | 0.0077 |  |  |  |
| GA_3 | IU_8 | 0.0075 |  |  |  |
| GA_4 | IU_10 | 0.0071 |  |  |  |
| GA_3 | IU_4 | 0.0047 |  |  |  |
| GA_4 | IU_7 | 0.0046 |  |  |  |
| GA_2 | IU_5 | 0.0044 |  |  |  |
| GA_3 | IU_1 | 0.0043 |  |  |  |
| GA_3 | IU_9 | 0.0033 |  |  |  |
| GA_3 | IU_2 | 0.0027 |  |  |  |
| GA_5 | IU_12 | 0.0023 |  |  |  |
| GA_6 | IU_10 | 0.0018 |  |  |  |
| GA_6 | IU_12 | 0.0011 |  |  |  |
| GA_7 | IU_7 | 0.0010 |  |  |  |
| GA_6 | IU_4 | 0.0009 |  |  |  |
| GA_4 | IU_5 | 0.0008 |  |  |  |
| GA_2 | IU_6 | 0.0001 |  |  |  |
| GA_5 | IU_6 | -0.0046 |  |  |  |
| GA_6 | IU_7 | -0.0098 |  |  |  |
| GA_5 | IU_3 | -0.0106 |  |  |  |

**Note:**

GA: generalized anxiety; IU: intolerance of uncertainty.

**Table S6** The directed edge weights between IU components and generalized anxiety symptoms in the CLPN of the high-anxiety group.

| **NodeOut** | **NodeIn** | **Value** | **NodeOut** | **NodeIn** | **Value** |
| --- | --- | --- | --- | --- | --- |
| **GA → IU** | | | **IU → GA** | | |
| GA_3 | IU_10 | 0.0579 | IU_11 | GA_5 | 0.0907 |
| GA_6 | IU_10 | 0.0557 | IU_4 | GA_2 | 0.0721 |
| GA_6 | IU_11 | 0.0405 | IU_4 | GA_3 | 0.0694 |
| GA_6 | IU_6 | 0.0393 | IU_11 | GA_6 | 0.0661 |
| GA_3 | IU_12 | 0.0390 | IU_11 | GA_1 | 0.0632 |
| GA_6 | IU_1 | 0.0382 | IU_11 | GA_3 | 0.0596 |
| GA_5 | IU_8 | 0.0330 | IU_11 | GA_2 | 0.0516 |
| GA_7 | IU_8 | 0.0306 | IU_10 | GA_4 | 0.0485 |
| GA_7 | IU_2 | 0.0286 | IU_10 | GA_3 | 0.0397 |
| GA_6 | IU_2 | 0.0280 | IU_1 | GA_5 | 0.0387 |
| GA_5 | IU_12 | 0.0241 | IU_11 | GA_7 | 0.0382 |
| GA_6 | IU_4 | 0.0182 | IU_2 | GA_7 | 0.0361 |
| GA_7 | IU_5 | 0.0155 | IU_4 | GA_6 | 0.0255 |
| GA_7 | IU_12 | 0.0153 | IU_11 | GA_4 | 0.0185 |
| GA_3 | IU_2 | 0.0150 | IU_4 | GA_4 | 0.0142 |
| GA_1 | IU_8 | 0.0066 | IU_1 | GA_4 | 0.0109 |
| GA_1 | IU_2 | 0.0064 | IU_4 | GA_1 | 0.0092 |
| GA_5 | IU_6 | 0.0055 | IU_10 | GA_2 | 0.0076 |
| GA_6 | IU_5 | 0.0039 | IU_10 | GA_5 | 0.0034 |
| GA_3 | IU_1 | 0.0034 | IU_2 | GA_4 | 0.0015 |
| GA_5 | IU_3 | -0.0047 | IU_6 | GA_6 | -0.0205 |
| GA_7 | IU_10 | -0.0120 | IU_5 | GA_5 | -0.0680 |
| GA_2 | IU_3 | -0.0121 |  |  |  |
| GA_2 | IU_6 | -0.0231 |  |  |  |

**Note:**

GA: generalized anxiety; IU: intolerance of uncertainty.

**
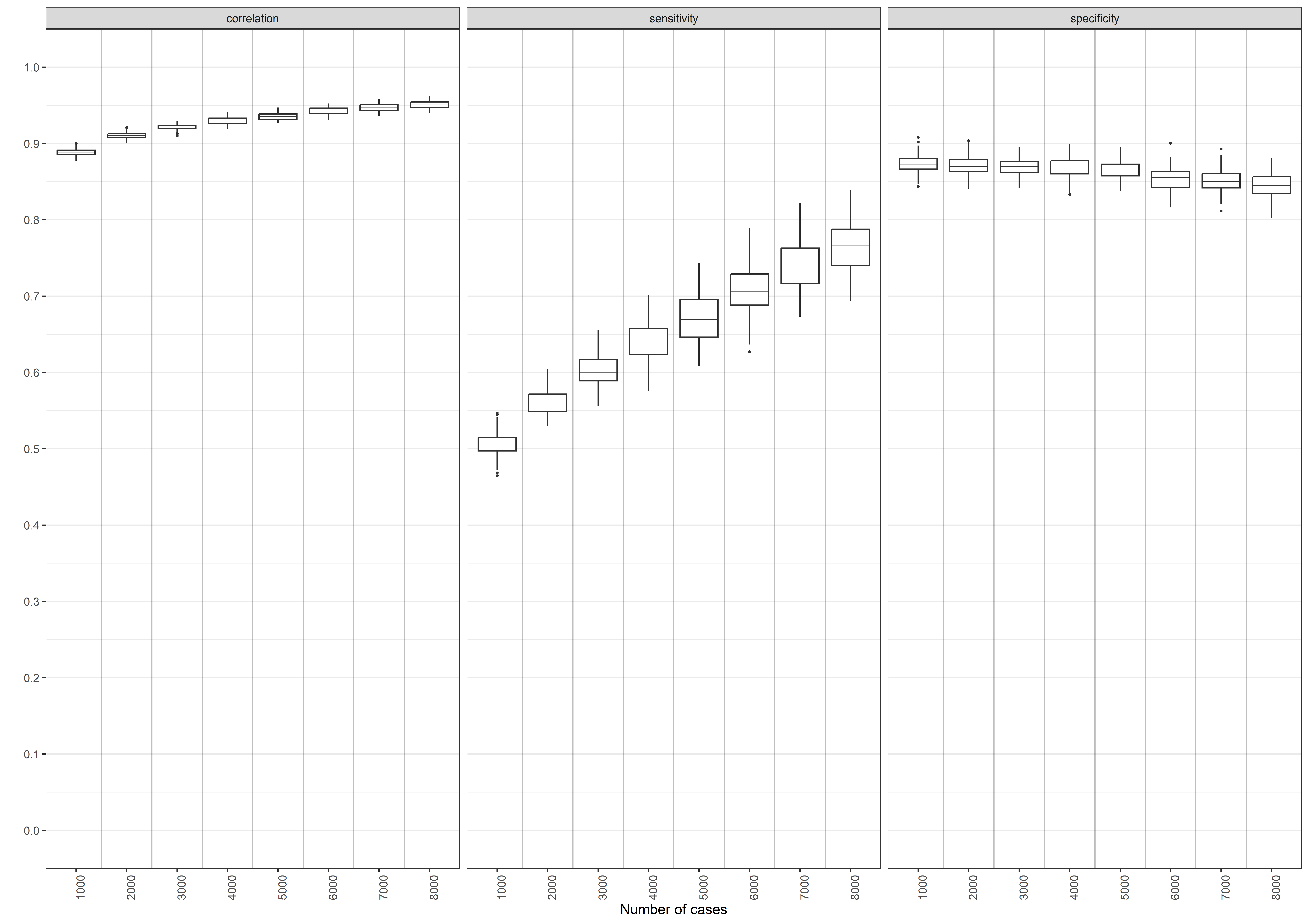
**

**Figure S1.** The power analysis simulation results of T1→T2 network.


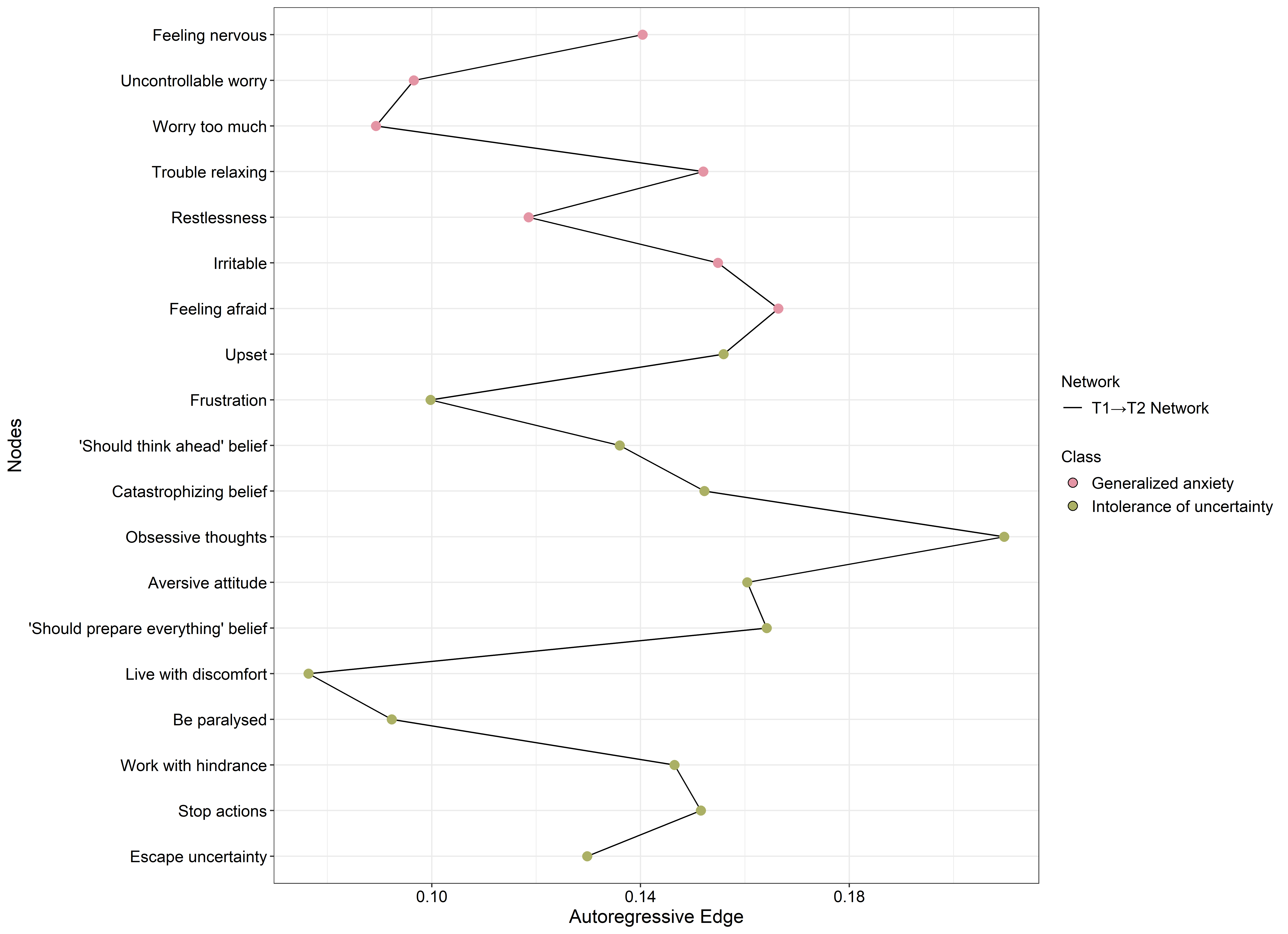


**Figure S2.** Autoregressive coefficients of each node.


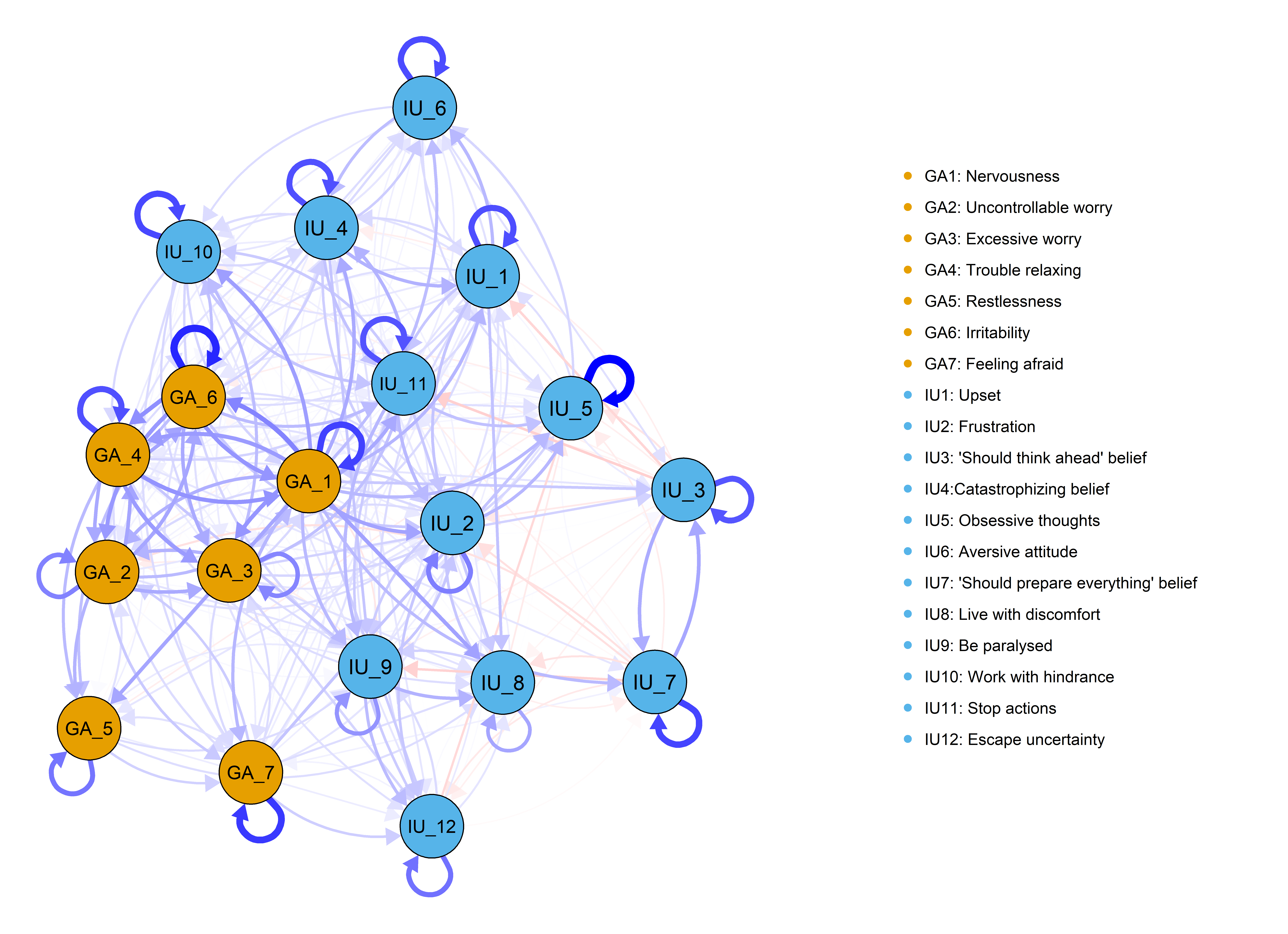


**Figure S3.** CLPN structures with auto-regressive edges (no threshold for edge weights). Arrows represent unique longitudinal relationships. Blue edges indicate positive relationships, and red edges indicate negative relationships. Thicker edges represent stronger relations. Covariates were excluded from the plot to ease visual interpretation.


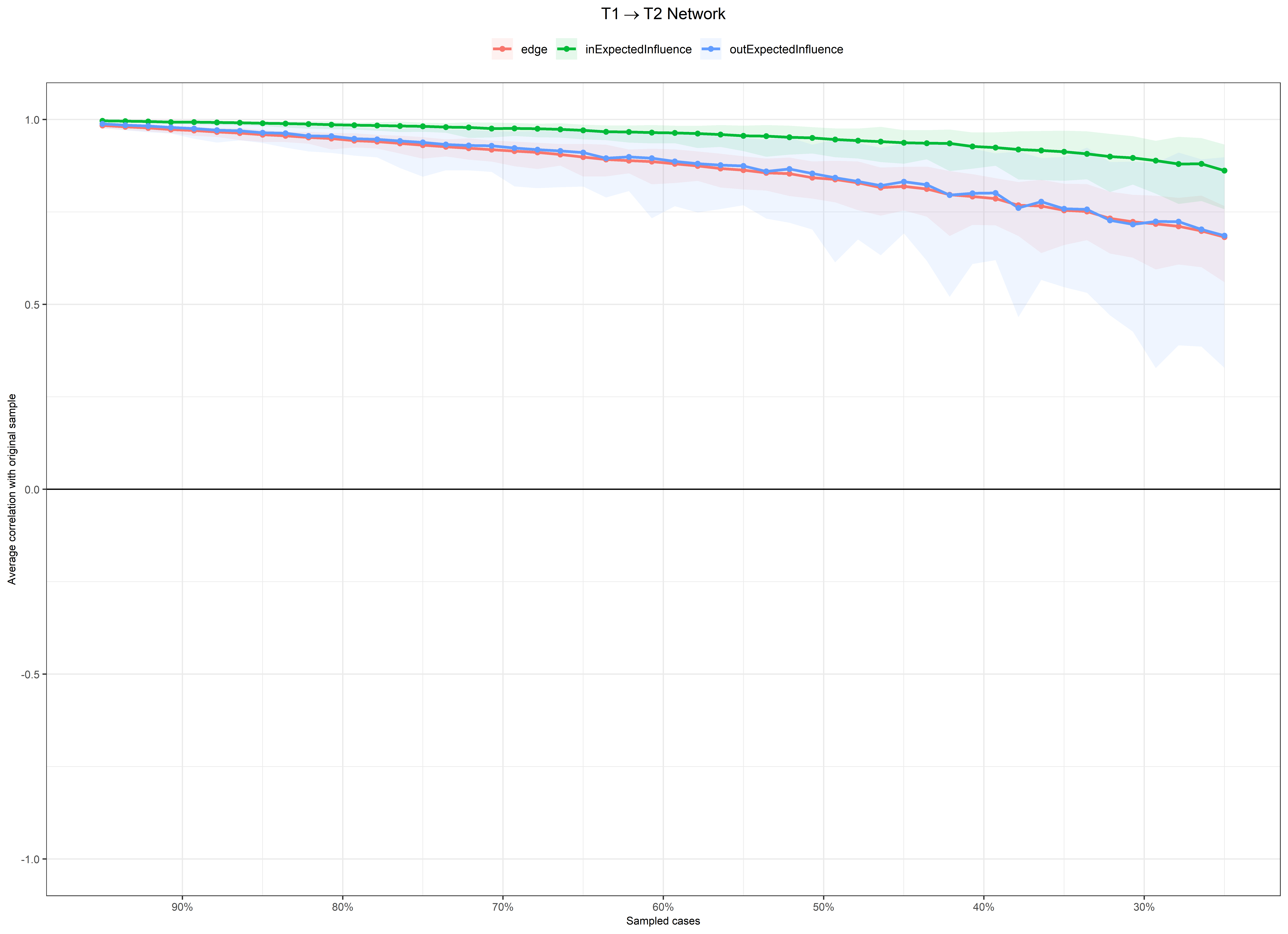


**Figure S4.** Case-dropping bootstrap test of edges and centrality indices for the CLPN. The x-axis indicates the percentage of cases of the original sample included at each step. The y-axis indicates the correlations between the centrality indices from the original network and the indices from the networks re-estimated after excluding increasing percentages of cases.


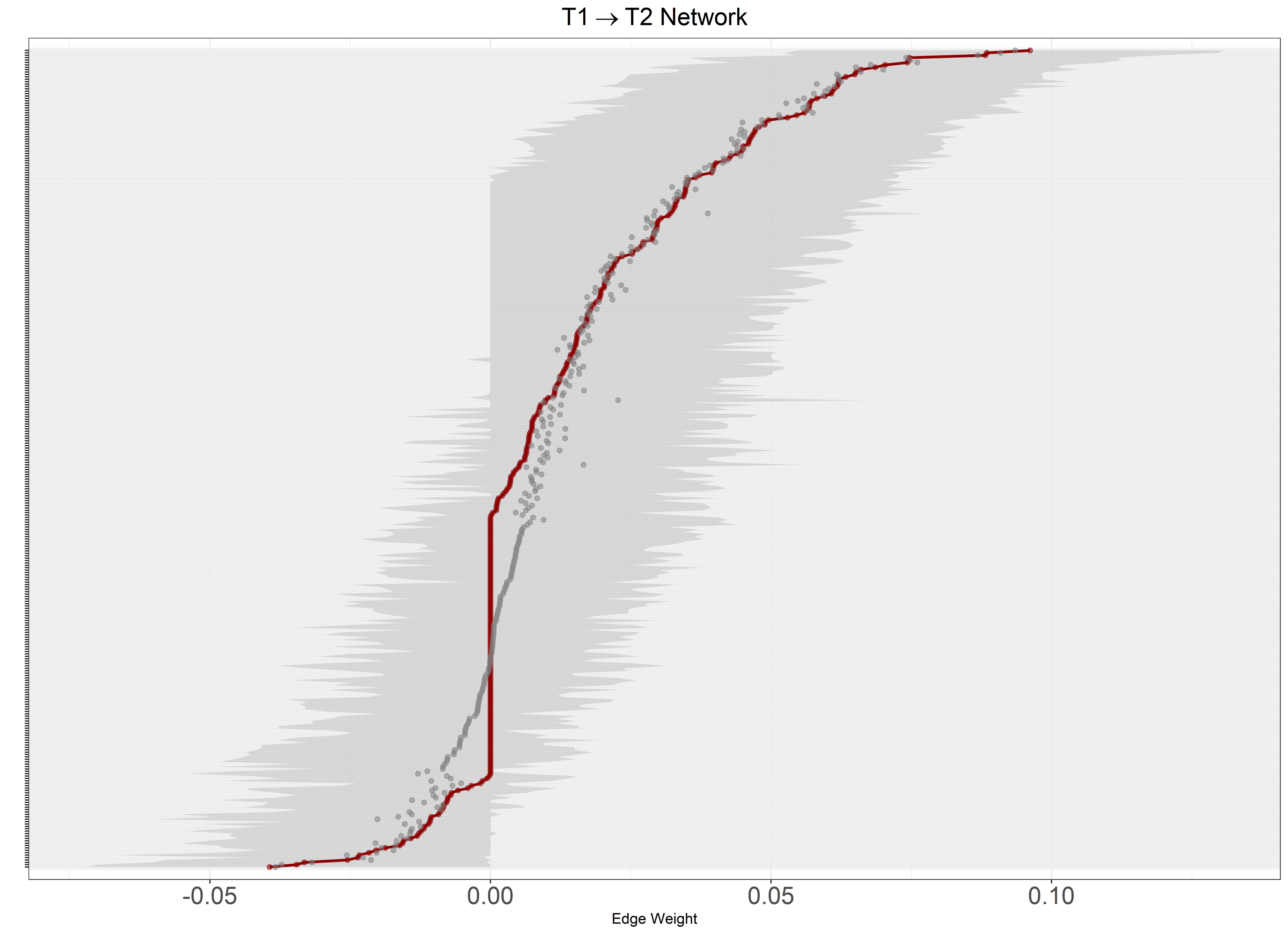


**Figure S5**. Nonparametric bootstrapped confidence intervals of estimated edges. The red line represents the estimated edge, while the dark area indicates the 95% bootstrap confidence interval.


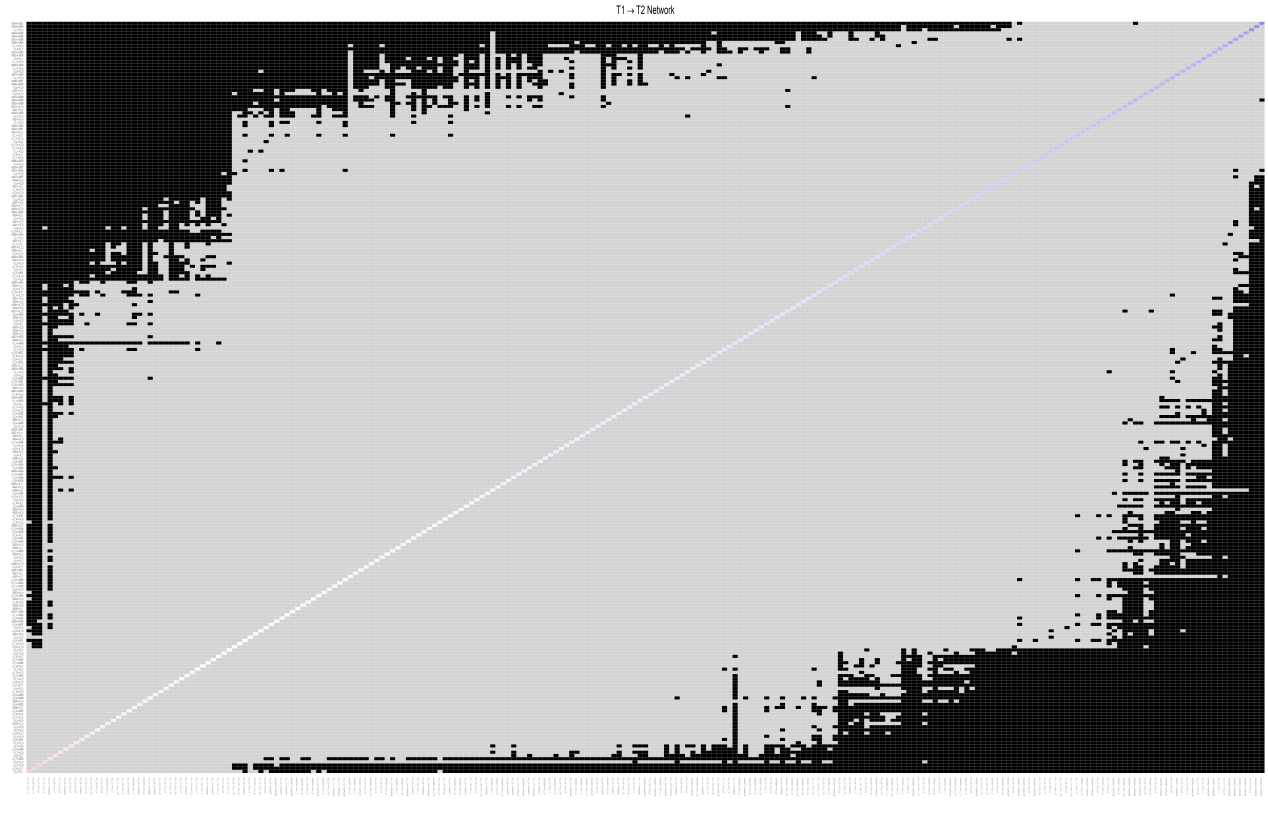


**Figure S6.** Bootstrapped difference tests for pairwise edge weights. The color of the boxes indicates whether edge weights differ significantly from each other (i.e., black) or do not differ significantly (i.e., grey).





**Figure S7.** Bootstrapped difference test for node centrality (i.e., in-EI and out-EI). The color of the boxes indicates whether oEI differ significantly from each other (i.e., black) or do not differ significantly (i.e., grey).


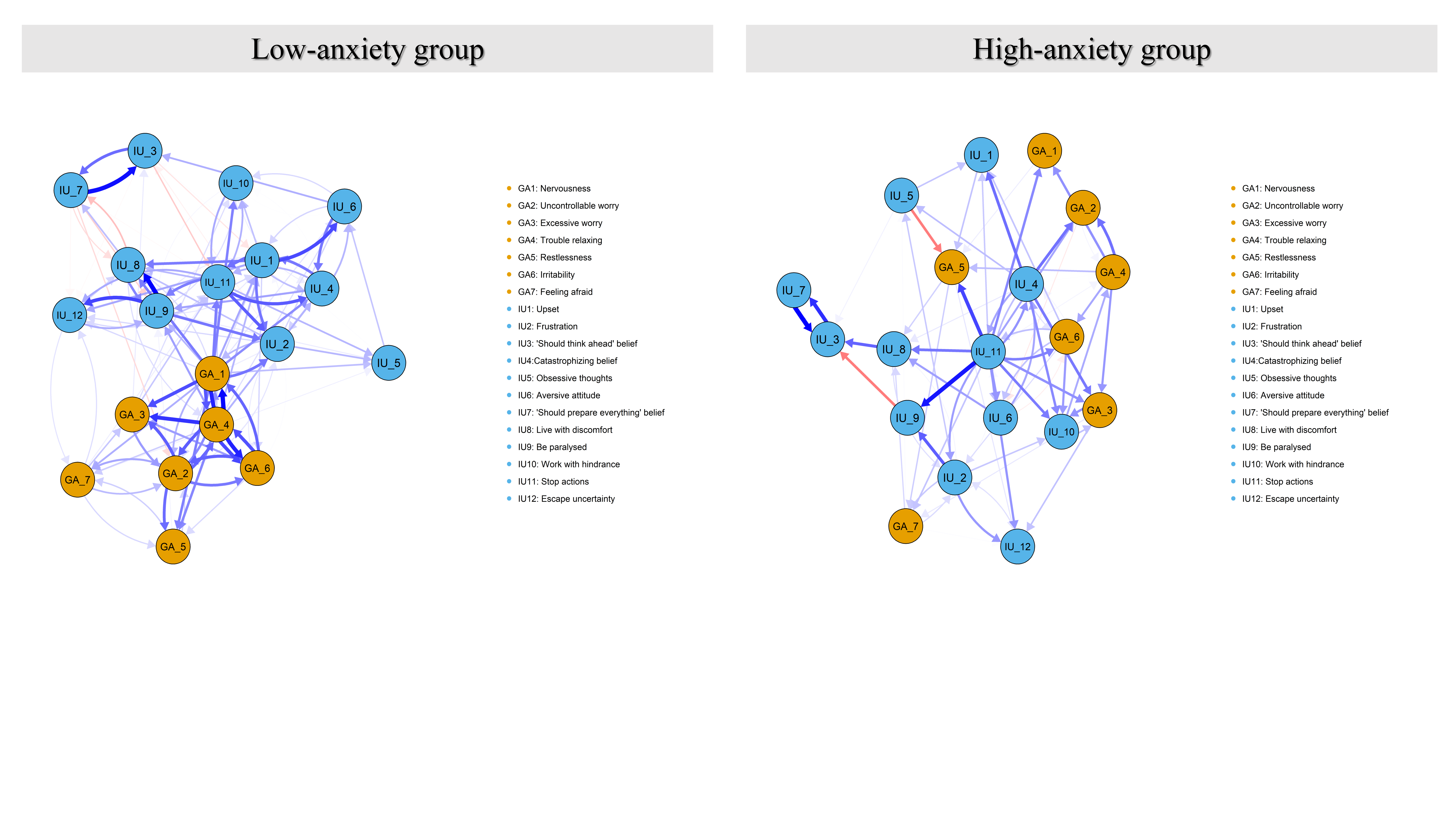
**Figure S8.** Plot for the CLPN (without auto-regressive edges) for low- and high-anxiety group. Edges depict cross‐lagged effects and arrows indicate the direction of prediction. Edge thickness reflects the strength of the effects. Only the edges ≥ 0.014 would be illustrated in the plot.

**
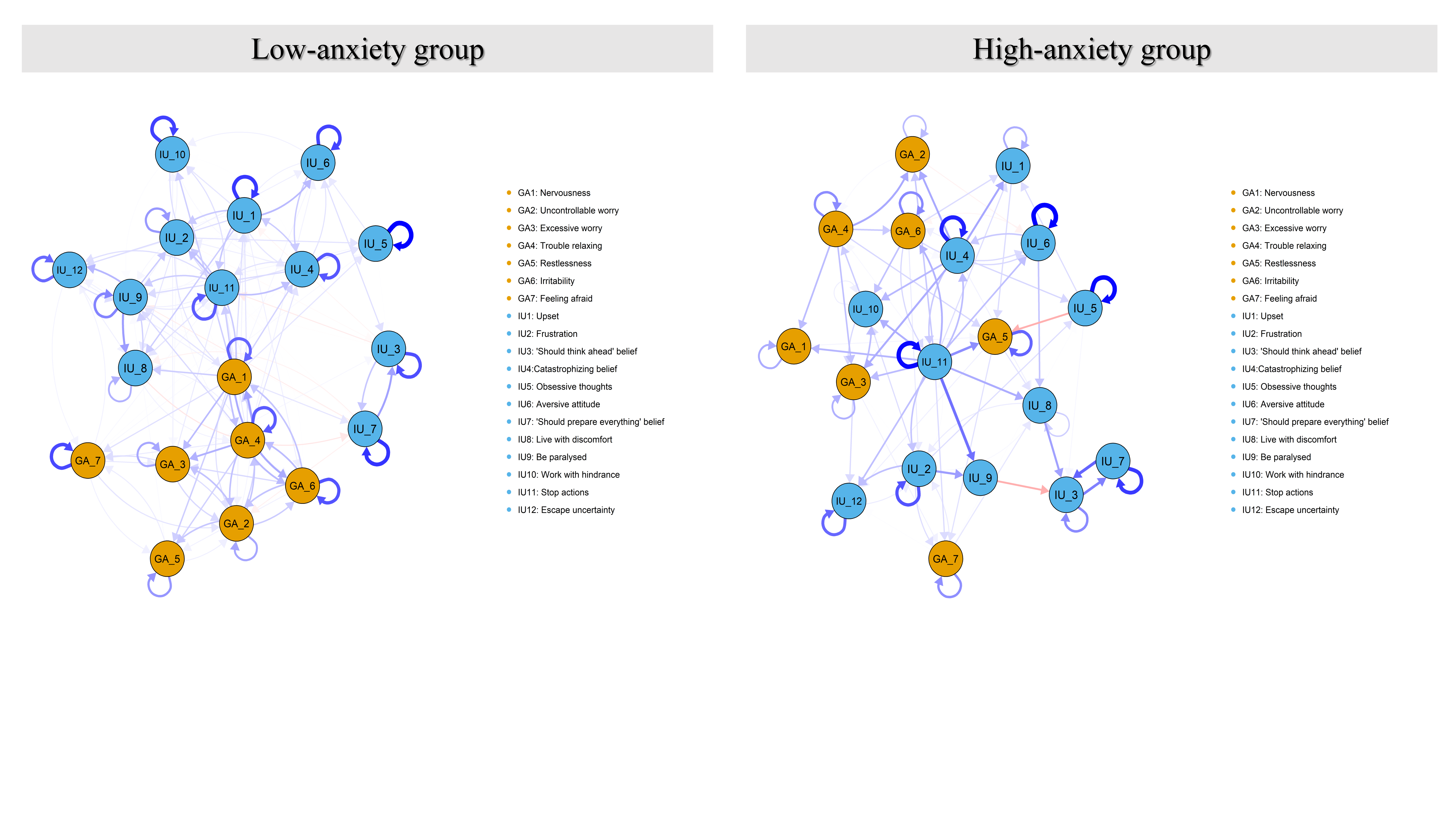
**

**Figure S9.** CLPN structures with auto-regressive edges (no threshold for edge weights) for low- and high-anxiety group. Arrows represent unique longitudinal relationships. Blue edges indicate positive relationships, and red edges indicate negative relationships. Thicker edges represent stronger relations. Covariates were excluded from the plot to ease visual interpretation.

**
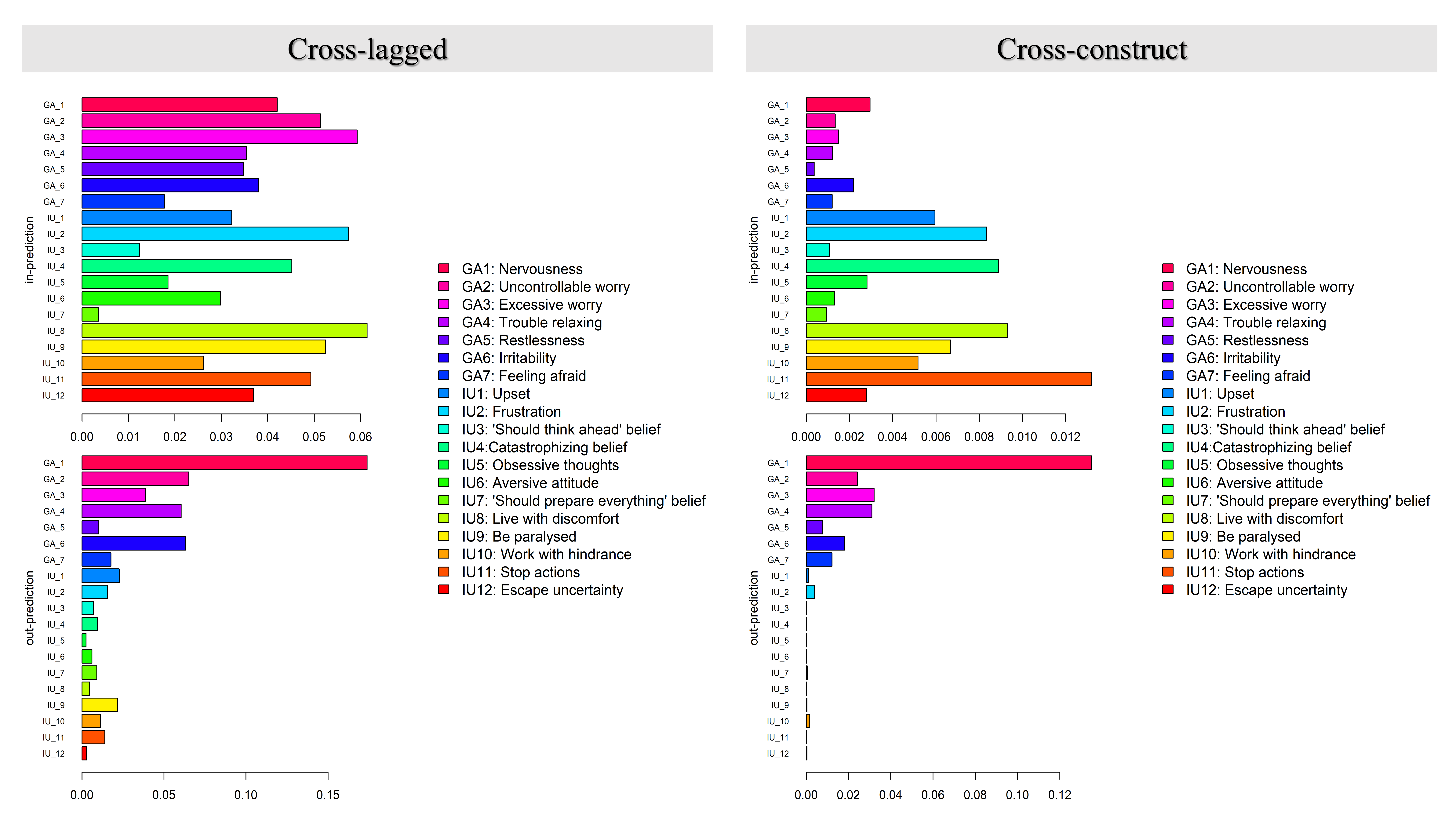
**

**Figure S10**. In-prediction and out-prediction for the cross-lagged and cross-construct of the CLPN for low-anxiety group.

**
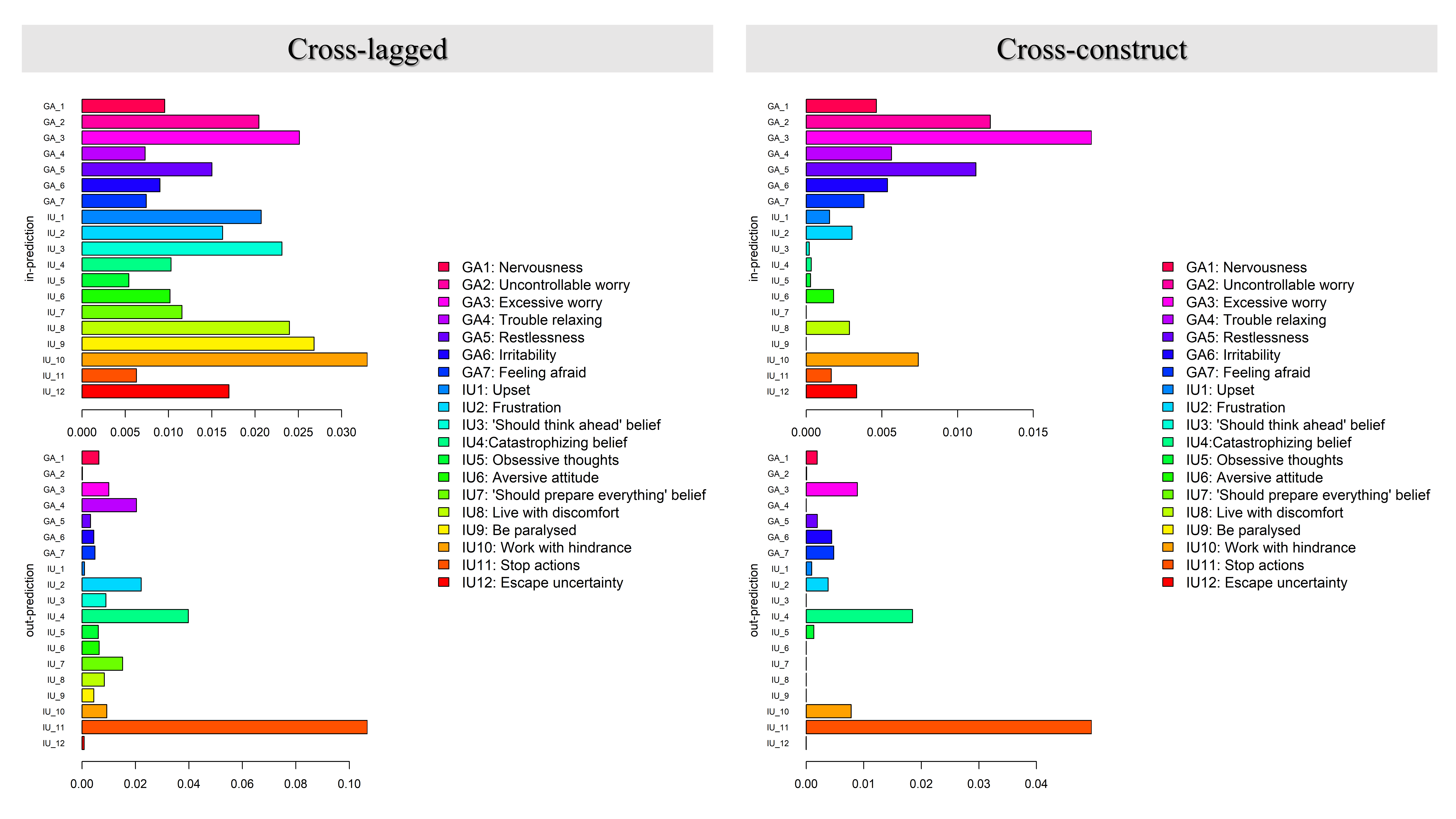
**

**Figure S11**. In-prediction and out-prediction for the cross-lagged and cross-construct of the CLPN for high-anxiety group.


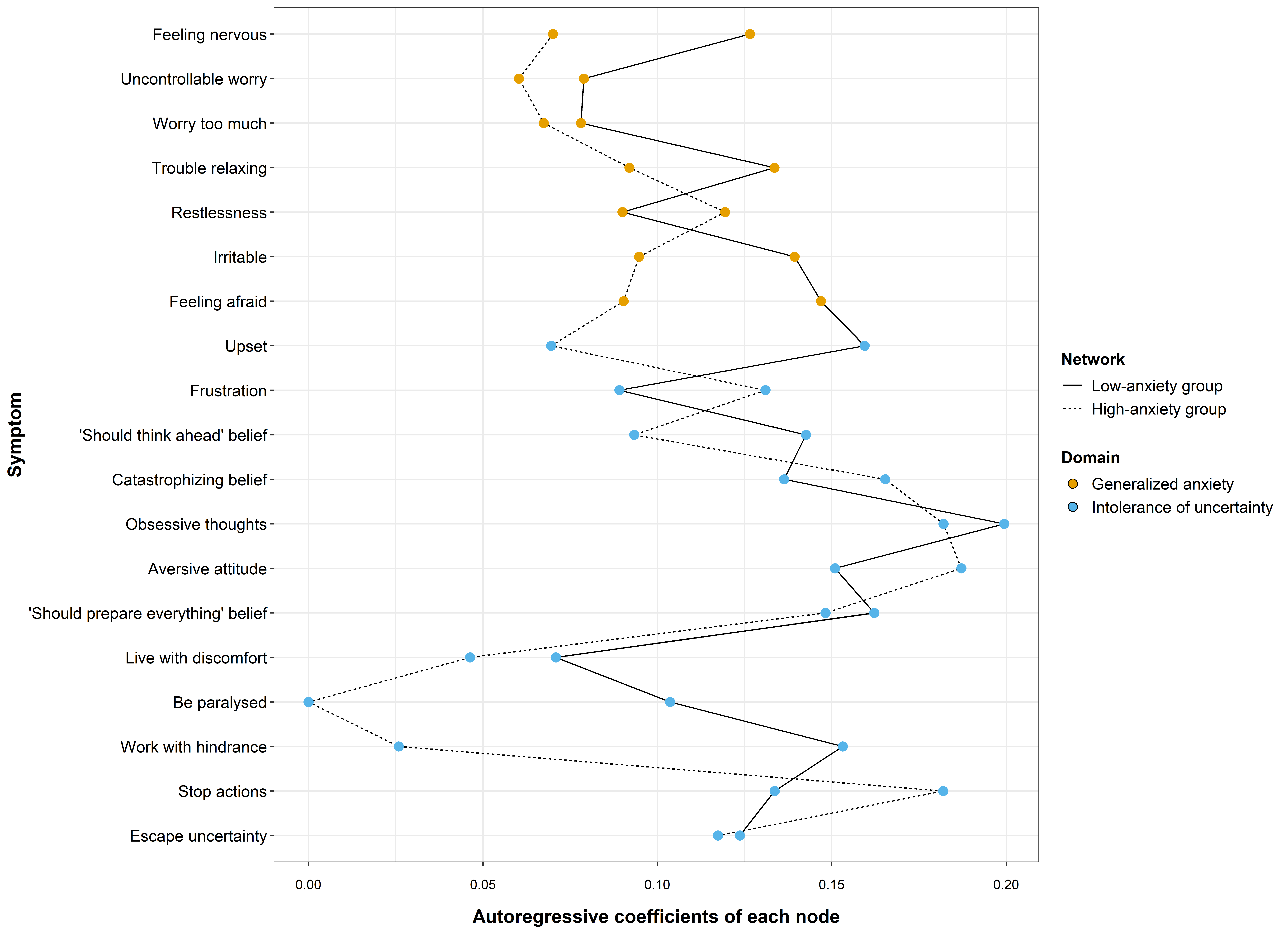


**Figure S12.** Autoregressive coefficients of each node for low- and high-anxiety group.

| **Low-anxiety group** | **High-anxiety group** |
| --- | --- |
| **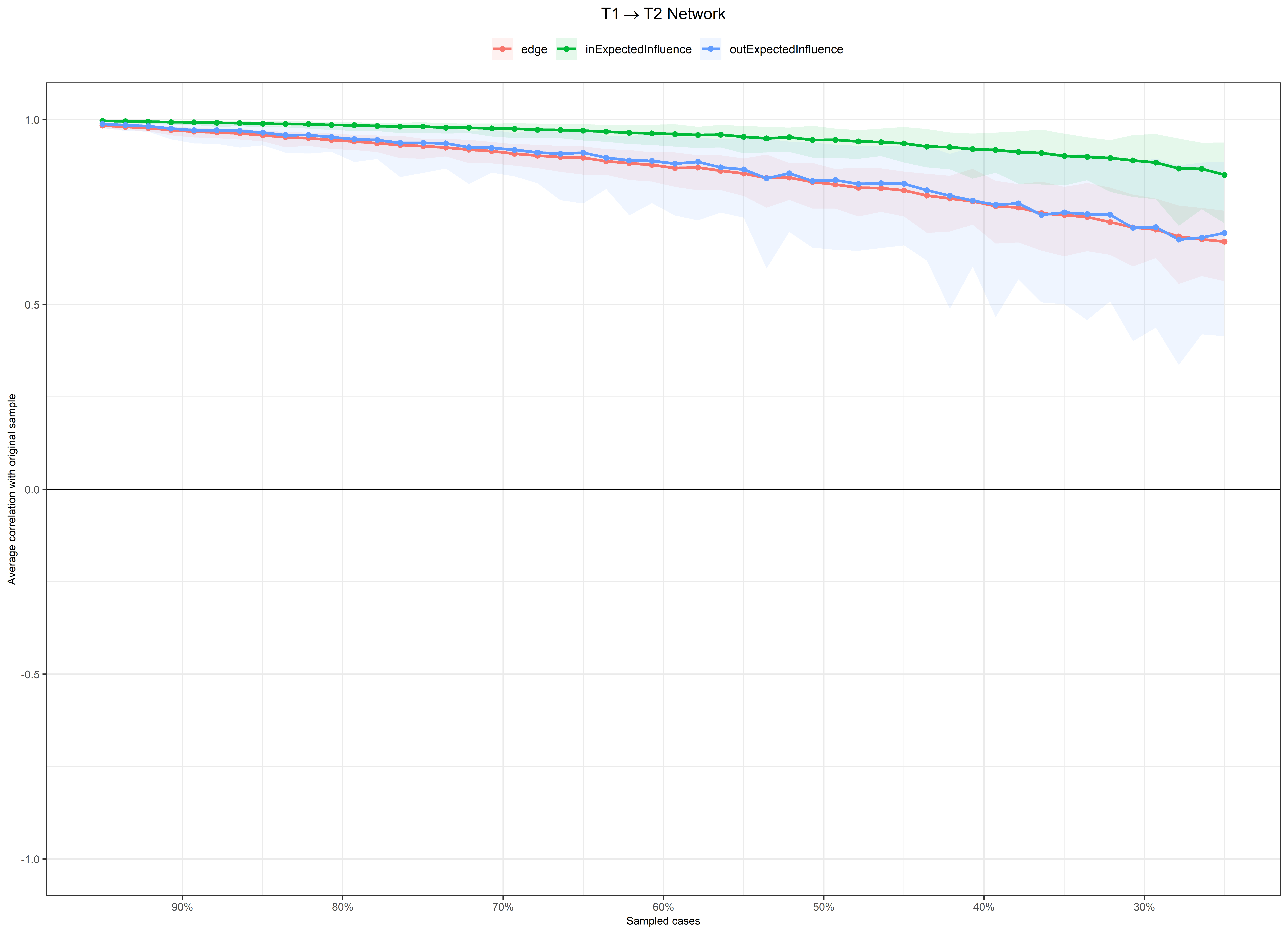** | **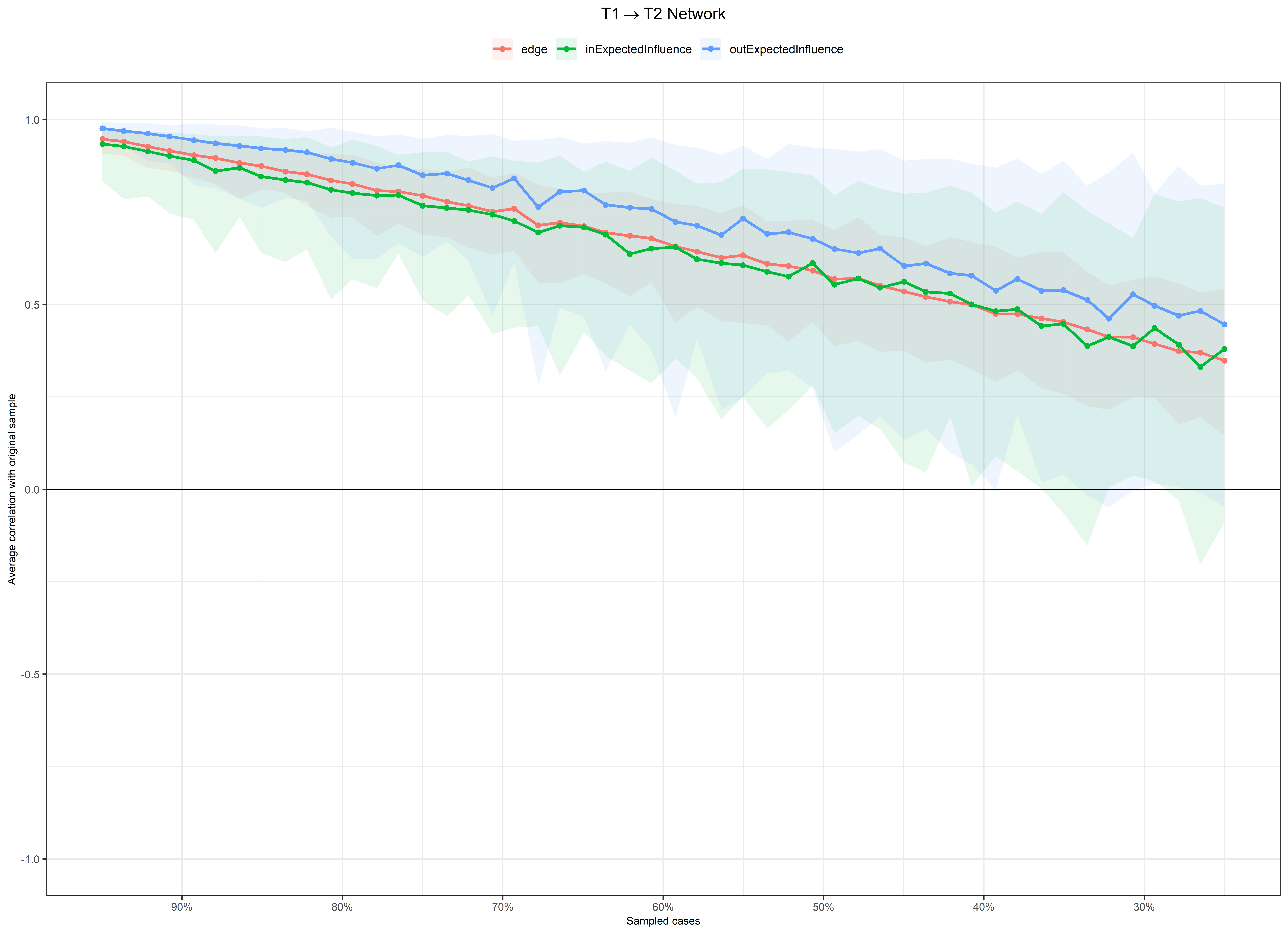** |

**Figure S13.** Case-dropping bootstrap test of edges and centrality indices for the CLPN. The x-axis indicates the percentage of cases of the original sample included at each step. The y-axis indicates the correlations between the centrality indices from the original network and the indices from the networks re-estimated after excluding increasing percentages of cases.

| **Low-anxiety group** | **High-anxiety group** |
| --- | --- |
| **** | **** |

**Figure S14**. Nonparametric bootstrapped confidence intervals of estimated edges. The red line represents the estimated edge, while the dark area indicates the 95% bootstrap confidence interval.

| **Low-anxiety group** | **High-anxiety group** |
| --- | --- |
| **** | **** |

**Figure S15.** Bootstrapped difference tests for pairwise edge weights. The color of the boxes indicates whether edge weights differ significantly from each other (i.e., black) or do not differ significantly (i.e., grey).

| **Low-anxiety group** | **High-anxiety group** |
| --- | --- |
| **** | **** |

**Figure S16.** Bootstrapped difference test for node centrality (i.e., in-EI and out-EI). The color of the boxes indicates whether oEI differ significantly from each other (i.e., black) or do not differ significantly (i.e., grey).
